# Satellite data reveal differential responses of Swiss forests to unprecedented 2018 drought

**DOI:** 10.1111/gcb.16136

**Published:** 2022-03-07

**Authors:** Joan Sturm, Maria J. Santos, Bernhard Schmid, Alexander Damm

**Affiliations:** ^1^ Department of Geography University of Zurich Zürich Switzerland; ^2^ Eawag, Swiss Federal Institute of Aquatic Science and Technology Dübendorf Switzerland

**Keywords:** environmental drivers, forest drought responses, NDWI, remote sensing, resilience, resistance, Sentinel‐2

## Abstract

Extreme events such as the summer drought of 2018 in Central Europe are projected to occur more frequently in the future and may cause major damages including increased tree mortality and negative impacts on forest ecosystem services. Here, we quantify the response of >1 million forest pixels of 10 × 10 m across Switzerland to the 2018 drought in terms of resistance, recovery, and resilience. We used the Normalized Difference Water Index (NDWI) derived from Sentinel‐2 satellite data as a proxy for canopy water content and analyzed its relative change. We calculated NDWI change between the 2017 pre‐drought and 2018 drought years (indicating resistance), 2018 and the 2019 post‐drought (indicating recovery), and between 2017–2019 (indicating resilience). Analyzing the data from this large natural experiment, we found that for 4.3% of the Swiss forest the NDWI declined between 2017 and 2018, indicating areas with low resistance of the forest canopy to drought effects. While roughly 50% of this area recovered, in 2.7% of the forested area NDWI continued to decline from 2018 to 2019, suggesting prolonged negative effects or delayed damage. We found differential forest responses to drought associated with site topographic characteristics and forest stand characteristics, and to a lesser extent with climatic conditions and interactions between these drivers. Low drought resistance and high recovery were most prominent at forest edges, but also on south‐facing slopes and lower elevations. Tree functional type was the most important driver of drought resilience, with most of the damage in stands with high conifer abundance. Our results demonstrate the suitability of satellite‐based quantification of drought‐induced forest damage at high spatial resolution across large areas. Such information is important to predict how local site characteristics may impact forest vulnerability to future extreme events and help in the search for appropriate adaptation strategies.

## Graphical abstract

We use the change of the normalized difference water index (NDWI) to detect which forest areas were damaged by the 2018 drought in Switzerland, and the conditions that lead to these effects. We found strongest linear relations between damaged forest and folded aspect, distance to the forest edge, elevation, and forest type. However, the direction and strength of the relationship varied based on the underlying time frame.

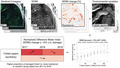

## 1 INTRODUCTION

The ongoing warming of the Earth's climate has been accompanied by an increased frequency of extreme events such as droughts, heatwaves, storms, or flooding (Baumgarten et al., 2019; Coumou & Rahmstorf, 2012; Miralles et al., 2019; Reichstein et al., 2013). Impacts of extreme events significantly affect ecosystems and humans at large (IPCC, 2021) through, for example, increased wildfires and air pollution, agricultural loss or green and blue water scarcity (Berdanier & Clark, 2018; Clark et al., 2016; Miralles et al., 2019; Puletti et al., 2019). Generally, the vulnerability of ecosystems increases when facing droughts (Allen et al., 2010, 2015; IPCC, 2014; Millar & Stephenson, 2015). However, it is not yet fully clear what effects these extreme events may have on ecosystems such as temperate forests in Switzerland and Central Europe. The extreme droughts of the 21st century (i.e., 2003, 2015, and 2018) are within the variability of drought events (Ionita et al., 2021) regarding precipitation deficits but unprecedented warming in the last millennium has led to an increase in frequency and magnitude of the compound climate extreme “hotter droughts” with disproportionate effects on ecosystems (Zscheischler & Seneviratne, 2017). After drought events, reduced primary productivity (Ciais et al., 2005), higher vulnerability to tree‐mortality, forest decline (Allen et al., 2010, 2015), shifts in species composition, and subsequent detrimental effects on ecosystem services (Rigling et al., 2013) have been observed to be dependent on site conditions (Lévesque et al., 2013; Rita et al., 2020). Nevertheless, it is still unclear what the long‐term consequences in terms of productivity and the tree's ability to resist pests and pathogens are (Ciais et al., 2005) and how these consequences influence global forest health (Allen et al., 2015). Furthermore, the need to extend from local field measurements to larger spatial scales (Lévesque et al., 2013) while maintaining fine‐scale spatial heterogeneity (Rita et al., 2020) for more generalizable results has been asserted.

Droughts, especially in combination with increased temperatures, represent a typical case of extreme events that become more intense stressors for temperate ecosystems and are likely to occur more frequently in the future (Horton et al., 2015; IPCC, 2014; Kornhuber et al., 2019; MeteoSchweiz, 2018). Drought types, that is, meteorological (Wilhite & Glantz, 1985), hydrological (Wilhite, 2000), agricultural (Dai, 2011), and ecological (Crausbay et al., 2017), may depend on different processes and cause different responses, making it necessary to clearly define which categorization is being used for a given drought event (Slette et al., 2019; Zang et al., 2020). Here, we use the concept of ecological drought for our quantification of its impacts. Ecological drought has been defined as “an episodic deficit in water availability that drives ecosystems beyond thresholds of vulnerability, impacts ecosystem services, and triggers feedbacks in natural and/or human systems” (Crausbay et al., 2017). Water availability deficits are driven partly by meteorological drought events or precipitation deficits that decrease soil moisture, runoff, and water storage. Further factors such as temperature, wind speed, radiation, and humidity alter the atmospheric evaporative demand (Miralles et al., 2019). Therefore water availability deficit affects plants directly by diminishing hydraulic conductance (Breshears et al., 2013) and indirectly through increased evapotranspiration (Teuling et al., 2013).

Ecological droughts may manifest themselves across different ecosystems, namely grasslands (Li et al., 2020; Zhang et al., 2018), tropical forests (Powers et al., 2020; Schwartz et al., 2019), temperate forests (Allen et al., 2010, 2015), taiga (Aakala & Kuuluvainen, 2011; Voltas et al., 2020), and tundra (Barber et al., 2000; Olson et al., 2018). Our understanding of ecological drought impacts comes from a combination of experimental and observational studies. In forests, there has been a recent recognition that these ecosystems substantially suffer from drought events (Allen et al., 2010, 2015; Brun et al., 2020; Buras et al., 2020; Cailleret et al., 2017; Schuldt et al., 2020; Senf et al., 2020), even though in absolute terms drought impacts in forests are less intense when compared to dryland ecosystems (O’Brien et al., 2017; Shekhar et al., 2020; Teuling et al., 2010; Wolf et al., 2013). Forest drought responses vary across temporal scales (Cavender‐Bares & Bazzaz, 2000). Short‐time responses (i.e., minutes to hours) include changes in stomatal closure (Jiang et al., 2019), altered energy balance (Sippel et al., 2018), a regulation of photosynthesis (Clark et al., 2016), and lowered hydraulic conductivity (Adams et al., 2017; Kukowski et al., 2013). Seasonal responses include phenological alterations, such as early wilting (Brun et al., 2020), leaf discoloration, leaf loss (MeteoSchweiz, 2018), growth reductions (Cailleret et al., 2017), and changes in resource allocation and repair mechanisms (Sippel et al., 2018). Long‐term responses (i.e., years to decades) or legacy effects include plant mortality (Berdanier & Clark, 2018; Buras et al., 2018; Clark et al., 2016; Wolf et al., 2016), an increased vulnerability of the forest to other stressors (Miralles et al., 2019), and reduced growth (stem and canopy) in recovering trees (Anderegg et al., 2015; Kannenberg et al., 2019, 2020; Schwalm et al., 2017). Droughts can affect multiple trees entire forest stands and landscape‐level processes (Byer & Jin, 2017; Rogers et al., 2018). These drought, responses include changed species abundances, interaction frequencies, and herbivory levels, that may even result in species turnover (Jiang et al., 2019; Sippel et al., 2018), or changed ecosystem functions such as primary productivity, erosion control, and fire suppression (Reichstein et al., 2013).

Climatic and location‐specific factors influencing forest vulnerability to drought include higher temperatures, which increase the atmospheric evapotranspirative demand and thus increase water stress (Allen et al., 2015; Buras et al., 2020). Forests in more exposed locations, for example, in ridges, are confronted with generally drier soils (Hawthorne & Miniat, 2018), often driven by higher wind and radiation exposure. Fragmentation plays an important role too, as forest edges may be more vulnerable to drought effects. At edges, both the micro‐climatic conditions and the larger canopy volumes result in higher evapotranspiration (Brun et al., 2020; Buras et al., 2018). The species‐specific water uptake depths of broadleaf trees (e.g., European beech, *Fagus sylvatica*) in contrast to conifers (e.g., Norway spruce, *Picea abies*) can shift to deeper soil levels and thus depleting water availability (Brinkmann et al., 2019). Higher forest density increases competition for water resources (Crowther et al., 2015; Sohn et al., 2016), whereas higher biodiversity allows functional compensation to stabilize forest ecosystems and increase resistance to disturbances (Anderegg et al., 2018; Isbell et al., 2015; Loreau et al., 2003).

Here, we aim at quantifying the effect of the abovementioned environmental influence factors on the drought response of Swiss forest ecosystems. With this, we respond to the need for comprehensive assessments to contribute to understanding whether our forests are resilient to anticipated climate change. More specifically, using Switzerland as a model system that provides many environmental gradients (Eilmann et al., 2009; Jolly et al., 2005; Rigling et al., 2013; Wolf et al., 2013), (i) we provide a remote sensing based assessment of drought effects based on canopy water content and (ii) we test the effect of the interplay of abiotic and biotic environmental drivers on forest drought responses. We took advantage of the naturally occurring sequence of pre‐drought, drought, and post‐drought conditions between 2017 and 2019 to assess the resistance, that is, capability to withstand disturbance (2017–2018), recovery, that is, capacity to reclaim performance after the disturbance (2018–2019), and resilience, that is, capacity to regain pre‐disturbance levels (2017–2019) of forest ecosystems to droughts (Albrich et al., 2020; Ingrisch & Bahn, 2018; Isbell et al., 2015; Lloret et al., 2011; Wagg et al., 2017). The combination of low precipitation and high temperature during the summer 2018 caused early leaf and needle discoloration, premature leaf shedding, canopy die‐back and tree mortality among many forests in Switzerland (Denzler, 2018, 2019; Medienmitteilung Basel Landschaft, 2018; Schuldt et al., 2020; SDA, 2019). The most affected tree species included Norway spruce and European beech (Schuldt et al., 2020), the two most common tree species in Switzerland occurring across 43.7% and 18.1% of the Swiss forest area, respectively. Previous studies focusing on the 2018 extreme drought event in Switzerland, compared it with the lower temperature drought in 1947 (Rathgeb et al., 2020), examined the geographical variability of its effects (Zappa et al., 2019), addressed its effects on the dominant tree species European beech (Baltensweiler et al., 2020) and forest health (Rohner et al., 2021). Across Central Europe, satellite‐based measurements of the Normalized Difference Vegetation Index have been used to assess impacts of the 2018 drought event (Brun et al., 2020; Buras et al., 2020; Schuldt et al., 2020). But we still lack knowledge on drought responses over large spatial extents while maintaining fine‐scale heterogeneity. In the present study we used Sentinel‐2 satellite data to derive a time series of the Normalized Difference Water Index (NDWI) as indicator of canopy water content (Gao, 1996).

We tested whether gradients of temperature, topography, fragmentation, and species heterogeneity affected forest drought responses. The underlying hypothesis is that more drought damages as measured by lower NDWI should occur at higher temperatures because of its effect on the evaporative demand, at ridges and along forest edges because of the effect of wind and radiation, for coniferous tree species (e.g., Norway spruce), and in mono‐specific or low‐species heterogeneity stands as more biodiversity is expected to provide more resilience. Together, we expect that tests of these hypotheses will further elucidate drought responses in temperate forests and increase our understanding of how such ecosystems respond to this increasingly more frequent stressor.

## 2 MATERIALS AND METHODS

### 2.1 A natural experiment—the 2018 summer drought in Switzerland

The summers of 2017, 2018, and 2019 were among the five warmest summers on record in Switzerland since measurements started in 1864 (Bastos et al., 2020; Brun et al., 2020; Buras et al., 2020; Gharun et al., 2020; Rohner et al., 2021; Schuldt et al., 2020) and were only surpassed by the summers of 2003 and 2015 (MeteoSchweiz, 2019; Orth et al., 2016). In 2017, precipitation in the summer half year (April–September) fell below the 1981–2010 norm (~ 800 mm) for most of Switzerland (MeteoSchweiz, 2017); thus, drier conditions are already part of our pre‐drought reference and could possibly cause an underestimate of the resistance and resilience of forests to the 2018 drought. The summer half year of 2018, with a nationwide precipitation of just above 500 mm, was the driest summer since 1962 (MeteoSchweiz, 2018). The summer of 2019 was similar to that of 2017, with average precipitation of ~740 mm (MeteoSchweiz, 2019).

**FIGURE 1:**
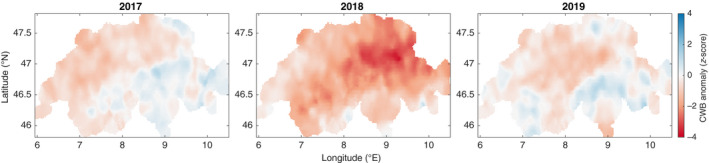
Climatic water balance (CWB) anomalies for the integrated periods of March to August 2017, 2018, and 2019 relative to the norm of 1981–2010 (data source: MeteoSwiss)

To capture the effects of the 2018 drought year on vegetation, we used the climatic water balance (CWB) as a measure of drought stress. This measure combines precipitation and temperature and, therefore, relates to water availability for plants (Senf et al., 2020; Vicente‐Serrano et al., 2010, 2014). We calculated CWB by subtracting the potential evapotranspiration, derived from mean monthly minimum and maximum temperatures (Hargreaves, 1994), from the monthly precipitation sum (Thornthwaite, 1984). We used monthly precipitation and temperature data products from MeteoSwiss (named RhiresM, TminM, and TmaxM in the MeteoSwiss data catalogue) at 2 km spatial resolution (Federal Office of Meteorology and Climatology, MeteoSwiss; https://www.meteoswiss.admin.ch/home/climate/swiss‐climate‐in‐detail/raeumliche‐klimaanalysen.html). We used the CWB values integrated from March to August for 2017, 2018, and 2019 to calculate CWB anomalies defined as the difference from the norm period 1981–2010 in terms of standard deviations of the norm period (z‐score). For example, the CWB anomalies for 2018 were more than two standard deviations below the norm period over large areas of Switzerland (Figure 1).

### 2.2 Using Sentinel‐2 data to assess forest condition

Copernicus Sentinel‐2 satellite data provide radiometric information of the visible to shortwave infrared (SWIR) in 13 distinct spectral bands with a spatial resolution of 10, 20, and 60 m (Drusch et al., 2012). We used three image mosaics comprising 11 Sentinel‐2 level‐1C (top‐of‐atmosphere reflectance) tiles to assess forest state in August 2017, 2018, and 2019. August was chosen since at this time of year there is a larger contrast between symptoms of environmental stress and the absence of senescence following the natural phenological cycle of Swiss forests (Zubler et al., 2014). Furthermore, August represents the peak time of the combined heat wave and drought in 2018 (MeteoSchweiz, 2018).

We applied an atmospheric correction to retrieve bottom‐of‐atmosphere reflectance (level‐2A) using the Sen2Cor processor (Louis et al., 2016) of the Sentinel Application Platform (SNAP ‐ ESA Sentinel Application Platform v6.0, http://step.esa.int). We also applied an empirical line‐based radiometric co‐registration of images (Ariza et al., 2018) to compensate possible inaccuracies in obtained reflectance values due to imperfect atmospheric characterizations over time and, thus, to further increase consistency of the time series. This procedure inter‐calibrates image radiometric information of a specific image of the time series considering a pre‐defined reference image (e.g., the first image of the time series). Therefore, a regression line is derived for corresponding reflectance values in two images (i.e., specific and reference image) that represent invariant targets, that is, materials whose reflectance is invariant or varies only slightly across time (e.g., artificial surfaces such as concrete or asphalt). The regression line is then used to adjust the reflectance values of the entire specific image considering the reference image. Therefore, we selected 11 to 29 pseudo‐invariant features for each tile (total 178 invariant locations) to estimate and correct wavelength‐dependent reflectance differences between years.

We masked clouds and their shadows using simple band thresholds determined by expert knowledge on pre‐processed reflectance data. Because clouds largely reflect light in the blue wavelength region, they are well distinguishable from low reflective forest areas in this wavelength region. The near infrared is well suited to distinguish cloud shadows from vegetation because vegetation shows substantially higher reflectance compared to cloud shadows. We then iteratively tested increments of 1% in reflectance in the blue and near infrared over different forest types to determine thresholds for clouds and cloud shadows. We disregarded all pixels with more than 5% reflectance in the blue (band 2 of Sentinel‐2, centered around 492 nm, bandwidth 66 nm, 10 m spatial resolution) and less than 15% reflectance in the near infrared (band 8 of Sentinel‐2, centered around 833 nm, bandwidth 106 nm, 10 m spatial resolution) for every single pre‐processed image. It should be noted that the applied cloud shadow threshold yields some false positives, for instance, vegetated pixels may be affected by cast shadow along mountain ridges. Besides clouds, artificial atmospheric effects (i.e., contrails) and other sensor errors generating false spectra can affect the analysis. Assuming that such errors only occur temporarily and manifested forest drought responses do not change substantially within one month, we calculated per pixel and spectral band the median from all pre‐processed and masked images acquired in August of a given year (1–7 images) to remove these effects and to work with one filtered spectral data cube per year.

The reflectance data obtained with the above procedure were used to calculate NDWI values (Gao, 1996) in the following way:

(1) $$NDWI=\;\frac{\left(\rho_{833}‐\rho_{1614}\right)}{\left(\rho_{833}+\rho_{1614}\right)}$$

where $\rho_{\left(\lambda \right)}$ stands for the top of canopy reflectance at a specific wavelength (λ; nm), which differ from the original definition using 860 nm and 1240 nm instead. The use of near infrared band 8 (centered around 833 nm, bandwidth 106 nm, 10 m spatial resolution) instead of band 8A (centered around 864 nm, bandwidth 21 nm, 20 m spatial resolution) comes at a cost of lower spectral resolution but has the advantages of a higher spatial resolution, and it has been successfully demonstrated that the such calculated NDWI is sensitive to water stress (Marusig et al., 2020). Higher spatial resolution also justifies the prioritization of band 11 (centered around 1614 nm, bandwidth 91 nm, 20 m spatial resolution resampled to 10 m) over band 10 (centered around 1374 nm, bandwidth 31 nm, 60 m spatial resolution). Furthermore, the whole SWIR is dominated by water absorption (Hill et al., 2019) and although the 1614 nm feature has much lower absolute reflectance values than the 1240 nm feature, it will only make the normalized difference more pronounced in comparison to the original NDWI. This adjusted NDWI was proposed by the European Drought Observatory and is sometimes called NDWI_1640_ (Wang et al., 2019), normalized difference infrared index (Wilson & Norman, 2018), or normalized difference moisture index (Jin & Sader, 2005).

It should be noted that the calculation of vegetation indices over mountainous regions can show artefacts stemming from an insufficient compensation of illumination geometries and atmospheric disturbances. In particular the actual irradiance over shaded hillslopes is difficult to estimate, resulting in wavelength‐dependent uncertainties that increase with shorter wavelengths, and propagate to retrieved bottom‐of‐atmosphere reflectance and derived vegetation indices (Damm et al., 2015). The identification of a robust index less sensitive to such topographical effects is essential to avoid misleading results. We compared the sensitivity of the NDWI to detect drought by comparing it to several indices commonly proposed to detect plant water stress, including the green‐red vegetation index (Tucker, 1979), the red chromatic coordinates (Gillespie et al., 1987), the normalized difference vegetation index (Tucker, 1979), and the enhanced vegetation index (Huete et al., 2002). We calculated these indices from the Sentinel‐2 imagery for August 2018 and compared mean and variance of the index values for a selection of forest pixels divided into three categories. These categories were as follows: (i) forest appearing healthy on flat terrain, (ii) forest visibly damaged (leaf discoloration) on flat terrain, and (iii) forest appearing healthy on steep slopes. A good index that would not be sensitive to topography should have similar values for the two categories (i) and (iii) but not (ii). This condition was best met by NDWI (Figure S1), likely because NDWI involves wavelengths in the near and short‐wave infrared that are less affected by wavelength dependent uncertainties common in the visible region (Guyot et al., 1992).

### 2.3 NDWI change from pre‐ to post‐drought as measure of forest drought response

To assess the response of forests to the 2018 drought year, we calculated NDWI changes (ratios) between (i) 2017 and 2018 as measure of resistance, (ii) 2018 and 2019 as measure of recovery, and (iii) between 2017 and 2019 as measure of resilience (Albrich et al., 2020; Ingrisch & Bahn, 2018; Isbell et al., 2015; Lloret et al., 2011; Wagg et al., 2017). Preceding dry conditions affecting the base condition (defined as August 2017) or recovery lasting longer than a year either from the drought 2018 or previous events were disregarded. To avoid autocorrelation and to aggregate the very large number of pixel data for further analysis, we then converted the continuous change measures into binary data using a 10% change as cutoff between “no change” and “strong change,” distinguishing between negative and positive changes. This binary approach divides pixels into non‐damaged and damaged forest, a differentiation important for our application purposes. The threshold allowed us to count the number of changed pixels relative to all pixels within a stratum containing pixels from a certain area or falling into the same category of an environmental variable (e.g., distance from forest edge). The percentage of positively or negatively changed pixels within a stratum was then used as the dependent variable measuring the response of forest to drought. The cutoff of 10% was chosen in the following way. We first flagged all forest pixels with leaf discoloration visible in high‐resolution orthophotos from August 2018 for a spatial subregion of ~10 km^2^ (Geoinformation Kanton Zürich, Orthofoto Sommer RGB/Infrarot 2018, https://maps.zh.ch/). Then, we iteratively tested increments of 1% change in NDWI until the resulting maps would cover the full extent of damage identified in the orthophotos.

Regarding interpretation, positive resistance would indicate an improved forest condition in the 2018 drought year compared to the pre‐drought year 2017 and should be rare, negative resistance indicates reduced condition in the drought year. Positive recovery indicates improved condition, while negative recovery indicates further damage in the post‐drought year 2019. For resilience, actually no change would be indicative of this response; however, using our measure, “over”‐resilience indicates that positive recovery “overcompensates” negative resistance whereas “under”‐resilience indicates that forest condition was still reduced in the post‐drought year compared with the pre‐drought year.

### 2.4 Validation

**FIGURE 2:**
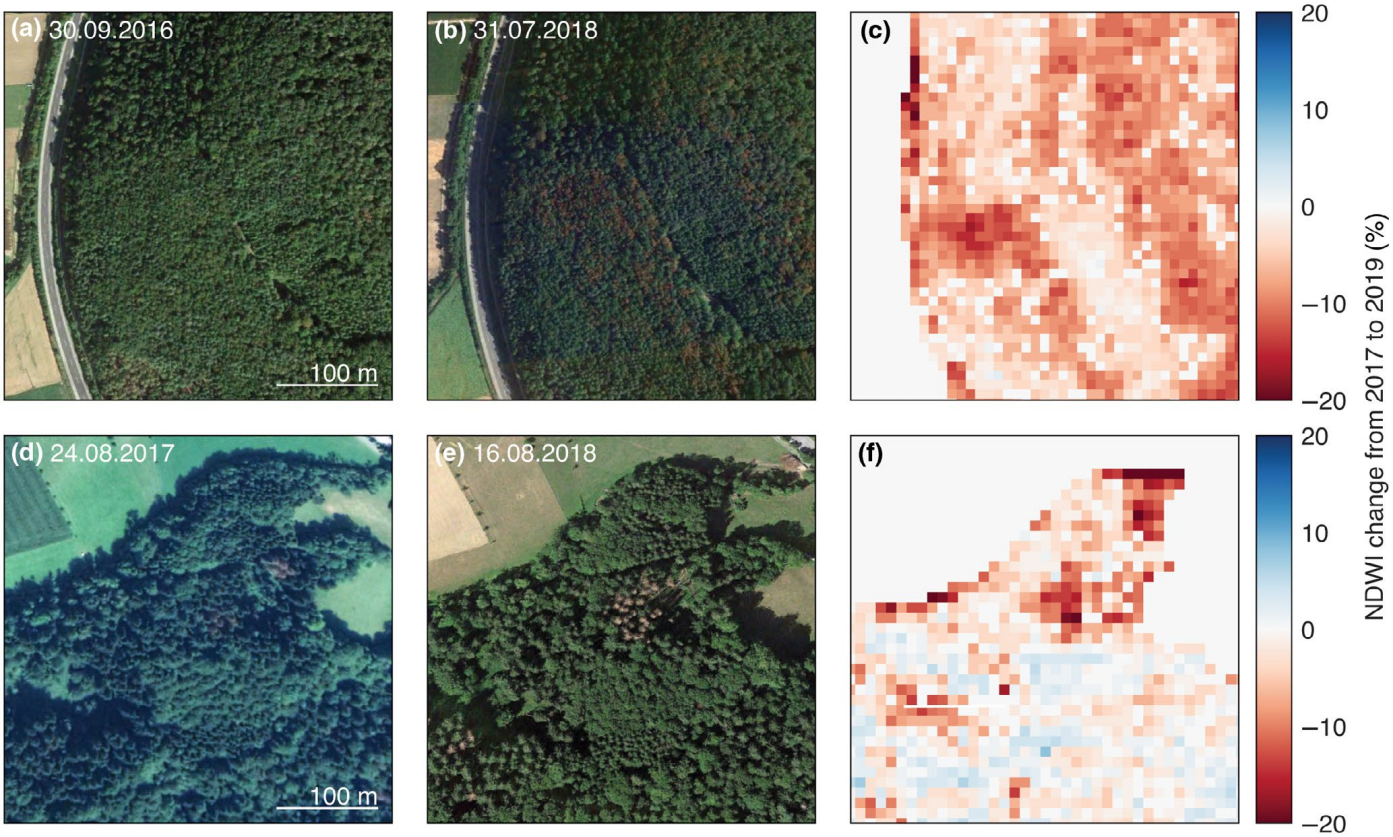
Subsets showing (a) healthy broadleaf trees in an RGB image at 47°44′23″N, 8°37′24″E (Map data: Google Earth), (b) the same trees with leaf discoloration in 2018 (Map data: Google Earth), (c) the percentual change in NDWI from 2017 to 2018 for the same location, as well as (d) healthy conifer trees in an RGB image at 47°16′27″N, 8°32′25″E (Map data: Google Earth, Image © 2021 Maxar Technologies), (e) the same conifers damaged in 2018 (Map data: Google), and (f) the percentual change in NDWI from 2017 to 2018 for these conifers

We validated the accuracy of the detected tree damage with high‐resolution satellite imagery acquired around the 2018 drought event and retrieved from Google Earth Pro (https://www.google.com/intl/en/earth/). We selected sample sites at regular intervals (~30 km) although missing summer imagery around the drought event resulted in gaps. Particularly in the alpine areas, imagery was scarce, whereas the northern and western part of Switzerland were widely covered. We identified damage in tree crowns that are related to canopy water content and thus NDWI (e.g., leaf discoloration for broadleaf trees (Figure 2b) or needle shedding for conifer trees (Figure 2e)) and digitized the extent of affected tree crowns. We identified and digitized tree crowns with no visible damage in close vicinity (max 4 km). In result, we collected 69 of these pairs of damaged and healthy trees for validation purposes. The accuracy of detected tree damage was assessed by the overall accuracy, the producer's accuracy, and user's accuracy from a standard confusion matrix.

### 2.5 Environmental variables potentially influencing drought response of forests

Switzerland offers large gradients of environmental conditions that may impact responses of forests to drought (Brun et al., 2020). We therefore assessed environmental variables that could potentially be the most important drivers of forest responses to drought. First of all, we assessed drought stress severity itself using climatic environmental variables. We used temperature and precipitation from March to August 2018 (Federal Office of Meteorology and Climatology, MeteoSwiss; https://www.meteoswiss.admin.ch/home/climate/swiss‐climate‐in‐detail/raeumliche‐klimaanalysen.html), as well as the above‐defined CWB anomaly (z‐score) (see section A natural experiment—the 2018 summer drought in Switzerland) to assess hotter (higher temperatures), drier (lower precipitation), and hotter and drier (low CWB z‐score) conditions. We aggregated monthly data products of the growing season (March–August) for mean temperature and total precipitation of the drought year (named TabsM and RhiresM in the MeteoSwiss data catalogue, 2 km grid size, resampled to fit Sentinel‐2 pixels) and compared the aggregated values with their corresponding values from 1981–2010 as percentual differences.

Next, we used data from the swissALTI^3D^ digital elevation model (DEM) (Federal Office of Topography, Swisstopo; www.swisstopo.admin.ch/en/geodata/height/alti3d.html) to derive elevation, slope, and folded aspect for each forested pixel of 10 × 10 m. We used the folded aspect to quantify to which degree slopes were facing south. The slope, aspect, and latitude were used to calculate the potential direct incident radiation (PDIR) (McCune, 2007; McCune & Keon, 2002). Additionally, we derived the topographic position index with an annulus (inner radius 150 m and outer radius 300 m) from the DEM (Weiss, 2001), where positive values indicate higher exposition.

We used the Swiss National Forest Inventory (NFI) forest mask with a spatial resolution of 1 x 1 m (Waser et al., 2015; WSL, 2020) to distinguish forested from non‐forested areas. The total forested area of Switzerland is 31% (BAFU, 2019). We applied a 20 m inward buffer to the forest mask to exclude mixed pixels due to the coarser spatial resolution of the satellite data compared with the NFI data and possible geometric distortions. We also used the forest mask to determine the closest distance of a forest pixel to the forest edge. We used the NFI tree type map (Waser & Ginzler, 2018; Waser et al., 2017) to distinguish broadleaf and coniferous trees and approximate species heterogeneity within 100 × 100 m moving neighborhoods. Overall, 28.6% of all Sentinel‐2 pixels covering Switzerland, not excluded due to clouds, topography, or buffering of the forest mask, represent forest and were further analyzed.

### 2.6 Statistical analysis

First, we used simple linear mixed models with ordinary least squares to assess the relationships between percentage affected forest pixels (resistance, recovery and resilience, for positive and negative changes; values were angular transformed [arcsine of the square root of the proportion] to ensure normality and homoscedasticity of residuals) and single environmental variables (Table 1) across 10 biogeographic regions in Switzerland (Federal Office for the Environment, FOEN; https://opendata.swiss/de/dataset/biogeographische‐regionen‐der‐schweiz‐ch). The 10 regions were used as blocks, that is, as a random term, to remove large‐scale geographic variation potentially masking overall effects of environmental variables. We divided the range of each environmental variable into 10 intervals to obtain similar stratifications for the separate analyses. Combined with the ten regions, this resulted in 100 strata to calculate percentage affected forest pixels, where “affected” refers to a greater than 10% change in NDWI (yielding six analyses per environmental variable, that is, for resistance, recovery or resilience combined with positive or negative changes). In other words, we binned the affected and all pixels according to the 10 biogeographic regions as well as 10 equally distributed bins of corresponding environmental variables, allowing the calculation of percentages by dividing the number of affected pixels by the number of all pixels in a bin (= stratum). Thus, our sample size for each model was 100 (10 × 10); except for a few cases where bins remained empty (e.g., high elevations in a region without tall mountains). Typically, the number of 10 × 10 m forest pixels (affected + unaffected) in each stratum or bin was several thousand or several ten thousand; and this number was used as a weighting variable. It should be noted that results without weighting according to number of pixels were very similar because all regions contained all or most intervals of all environmental variables. The corresponding binned data (affected and all pixels as well as mean NDWI changes per stratum or bin) are available in the dataset deposited online (Sturm, 2022). We fit linear models (lm) with the factor region followed by the environmental variable (10‐level continuous variate) as lm(y ~ region +environmental variable) in RStudio (RStudio Team, 2018) to obtain slopes and *R* ^2^ values for each environmental variable corrected for regions. Note that this method is equivalent to the alternative mixed‐model formulation lme(y ~ environmental variable, random=1|region) (Schmid et al., 2017). The corrected R^2^ values were calculated by dividing the sum of squares (SS) of the environmental variable by the sum of the SS of the environmental variable and the SS of the residuals. The slope of the regression line along the 10 intervals indicates the direction of the correlation, and the coefficient of determination (*R* ^2^) expresses the goodness‐of‐fit of the linear model. Nonsignificant correlations (*p* ≥ .05) were disregarded. For illustration, three examples are shown in the supplementary information (Figures S2–S4). In addition to analyzing the percentages of pixels with >10% change between years, we also analyzed the mean change, that is, mean resistance, mean recovery, and mean resilience directly (Figure S5), and to verify the used 10% threshold we also tested 5% (Figure S6) and 15% (Figure S7) cutoff values. Because all these analyses yielded similar results, we focus our presentation on the analysis with 10% cutoff values.

**TABLE 1:** Environmental variables with their units, range, and the interval size applied in the simple linear mixed models

Environmental variable	Unit	Range (Min, Max)	Interval size
Temperature anomaly to norm	%	100, 180	8
Precipitation anomaly to norm	%	45, 120	7.5
CWB anomaly (z‐score)	Standard deviations	–3.8, 1.1	0.49
Elevation	m a.s.l.	190, 2500	231
Slope	°	0, 90	9
Folded aspect	°different from North	0, 180	18
Potential direct incident radiation	MJ cm^−2^ yr^−1^	0.23, 0.52	0.029
Topographic position index	–	–3000, 3000	600
Distance to forest edge	m	0, 1500	150
Species heterogeneity	(% of broadleaf tree)^2^	0, 1500	150
Forest type	% of broadleaf tree	0, 100	10

**FIGURE 3:**
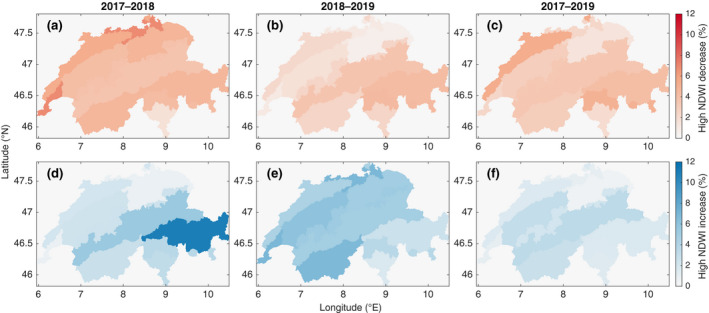
Choropleth maps of ten biogeographic regions (data source: FOEN) across Switzerland colored according to the proportion of forest areas with a change in NDWI ≤ –10% (upper row) and ≥10% (lower row) for the period from 2017 to 2018 (a and d, respectively), 2018 to 2019 (b and e), and 2017 to 2019 (c and f)

To obtain a conditional analysis for recovery, we repeated the above analyses for the subsets of pixels that had only positive, only negative, or only no changes >10% from 2017 to 2018. This allowed us to test if, for example, positive recovery was generally following low resistance, that is, affecting pixels that had lost >10% in NDWI from 2017 to 2018, or if frequently pixels would be negatively affected in the two yearly intervals in a row.

Second, we used more complex linear mixed‐effects models to assess the effect of the most significant environmental variables from the univariate analysis described above as well as their interactions. However, to avoid overfitting with too many strata (bins), we made an aggregated multidimensional table using three intervals for each of five chosen environmental variables and the 10 regions (see description below). With this reduction of the dataset from millions of pixels to 3^5^ * 10 = 2430 strata or bins—of which 2108 were non‐empty and for which the percentage affected pixels could be calculated—the data could be handled by lme() to test significances and lm() to obtain effect sizes. For the latter we used percent SS, which correspond to increments of multiple *R* ^2^ as terms are fitted in sequence. Note that changing the sequence of terms had very little influence because the aggregated table resulted in high orthogonality among explanatory variables even when using number of pixels per stratum or bin as weighting variable. We used three intervals per environmental variable to allow for detection of potential non‐linearities and, in cases where we detected these, we accounted for them using contrasts (Schmid et al., 2017). The intervals for the environmental variables were the following: for *elevation*: lowland (0–800 m a.s.l.), foothills (801–1600 m a.s.l.), Alps (>1600 m a.s.l.); for *folded aspect*: north (0–60°), west or east (61–120°), south (121–180°); for *topographic position index*: valley‐bottoms (<–1 standard deviation), slopes (≥–1 and ≤1 standard deviation), ridgetops (>1 standard deviation) (Weiss, 2001); for *distance to forest edge* (after applying a 20‐m buffer): edge (0–20 m), intermediate (21–50 m), interior (>50 m); for *forest type*: coniferous (0–20% broadleaf), mixed (21–79% broadleaf), broadleaf (80–100% broadleaf). The percentage of affected pixels per stratum was again angular transformed and the total number of pixels used as weighting variable. As in the simple linear mixed models, region was considered as random factor in the complex linear mixed models. The following final linear models were fitted (lme to assess significance, lm to obtain effect sizes in terms of % of SS):

- lme(ang(y) ~ ELE + ASP + TOP + DIS + COM + ELE:ASP + ELE:TOP + ELE:DIS + ELE:COM + ASP:TOP + ASP:DIS + ASP:COM + TOP:DIS + TOP:COM + DIS:COM, random=~1|REG, weights=varFixed(~1/N))
- lme(ang(y) ~ ele + ELE + asp + ASP + top + TOP + dis + DIS + com + COM + ele:asp + ELE:asp + ele:ASP + ELE:ASP + ele:top + ELE:top + ele:TOP + ELE:TOP + ele:dis + ELE:dis + ele:DIS + ELE:DIS + ele:com + ELE:com + ele:COM + ELE:COM + asp:top + ASP:top + asp:TOP + ASP:TOP + asp:dis + ASP:dis + asp:DIS + ASP:DIS + asp:com + ASP:com + asp:COM + ASP:COM + top:dis + TOP:dis + top:DIS + TOP:DIS + top:com + TOP:com + top:COM + TOP:COM + dis:com + DIS:com + dis:COM + DIS:COM, random=~1|REG, weights=varFixed(~1/N))
- lm(ang(y) ~ REG + ele + ELE + asp + ASP + top + TOP + dis + DIS + com + COM + ele:asp + ELE:asp + ele:ASP + ELE:ASP + ele:top + ELE:top + ele:TOP + ELE:TOP + ele:dis + ELE:dis + ele:DIS + ELE:DIS + ele:com + ELE:com + ele:COM + ELE:COM + asp:top + ASP:top + asp:TOP +ASP:TOP + asp:dis + ASP:dis + asp:DIS + ASP:DIS + asp:com + ASP:com + asp:COM + ASP:COM + top:dis + TOP:dis + top:DIS + TOP:DIS + top:com + TOP:com + top:COM + TOP:COM + dis:com + DIS:com + dis:COM + DIS:COM, weights=N))

where REG = region as a random factor with 10 levels, ELE = elevation as a fixed factor with 3 levels, DIS = distance to forest edge as a fixed factor with 3 levels, ASP = folded aspect as a fixed factor with 3 levels, TOP = topographic position index as a fixed factor with 3 levels, COM = forest type as a fixed factor with 3 levels, N = total number of pixels per stratum. In (iii), lower‐case terms represent contrasts as follows: ele = lowland versus foothills and Alps, asp = north versus south, top = slopes versus valley‐bottoms and ridge‐tops, dis = forest edge versus non‐edge, and com = broadleaf trees versus mixed and coniferous trees. Note that when a contrast term is followed by the factor, the latter provides a test for remaining differences among levels once the differences due to the contrast have been explained. For example, ele + ELE partitions the factor ELE (2 degrees of freedom (DF)) into the contrast lowland versus foothills and Alps (ele, 1 DF) and the remaining difference foothill versus Alps (ELE fitted after ele, 1 DF). Thus, the models (i)–(iii) all fit the same number of parameters, namely 51 (see Table S1). Fitting main effects and all 2‐way interactions for the five environmental terms, we could keep the number of fitted parameters small relative to the number of data values (more than 40 data points per estimated parameter, that is, DF of residual 2048). As for the single analyses above, we also compared the results of the complex analysis using percentages of pixels with >10% changes between years with a complex analysis with the mean changes in NDWI between years directly, that is, mean resistance, mean recovery, and mean resilience (Table S2). Again, this analysis yielded similar results and we thus again focus our presentation on the analysis with 10% cutoff values.

## 3 RESULTS

### 3.1 Forest resistance, recovery, and resilience to the 2018 drought

#### 3.1.1 Forest resistance (NDWI changes ≥10% from 2017–2018)

We found that NDWI decreased by ≥10% during the drought event in 2018 for 4.29% of the Swiss forest area. The highest proportions of decreasing NDWI values were found in the North and Southwest (6.86%, Figure 3a). The lowest proportion (1.63%) was found in the South. For 3.76% of the forest area, NDWI values increased by ≥10%. The highest proportion of areas with increasing NDWI from 2017–2018 (here referred to as “over”‐resistance) were found in the southeastern part of Switzerland (11%, Figure 3d). The smallest proportion of increasing NDWI values (0.79%) was found in the Northeast.

#### 3.1.2 Forest recovery (NDWI changes ≥10% from 2018–2019)

After the drought event, 2.7% of the forested area in Switzerland experienced a decrease in NDWI of ≥10% (here referred to as “negative” recovery), with the highest proportions found in the southeastern regions (4.31%–4.42%). Least NDWI decrease was found in the Northeast, where 0.6% of the forest area was strongly affected. Of the Swiss forest area, 4.63% showed positive recovery with an increase in NDWI of ≥10%. The Southeast showed the lowest proportions of increasing NDWI values. Forest recovery in the strict sense refers to recovery from a previous loss, but the above numbers include cases of previous losses, gains or <10% changes. In section Recovery of forest areas after negative, neutral, or positive resistance, we, therefore, analyze resistance separately for these three scenarios.

#### 3.1.3 Forest resilience (NDWI changes ≥10% from 2017–2019)

Looking at the entire drought event including the pre‐ and post‐drought year, we found 3.54% of the forest area with decreasing NDWI values of ≥10%, indicating continuing negative effects of the drought‐year 2018 into the post‐drought year 2019 (here referred to as “under”‐resilience). The highest proportion (5.36%) was found in the Northwest, whereas lowest proportions of strong NDWI decreases were found in the Northeast (1.4%) and the South (1.93%). Generally, 2.15% of forest areas showed strongly increasing NDWI from 2017 to 2019 (here referred to as “over”‐resilience) with 3.37% along the northern side of the Alps.

### 3.2 Relation between climatic variables and forest drought response

**FIGURE 4:**
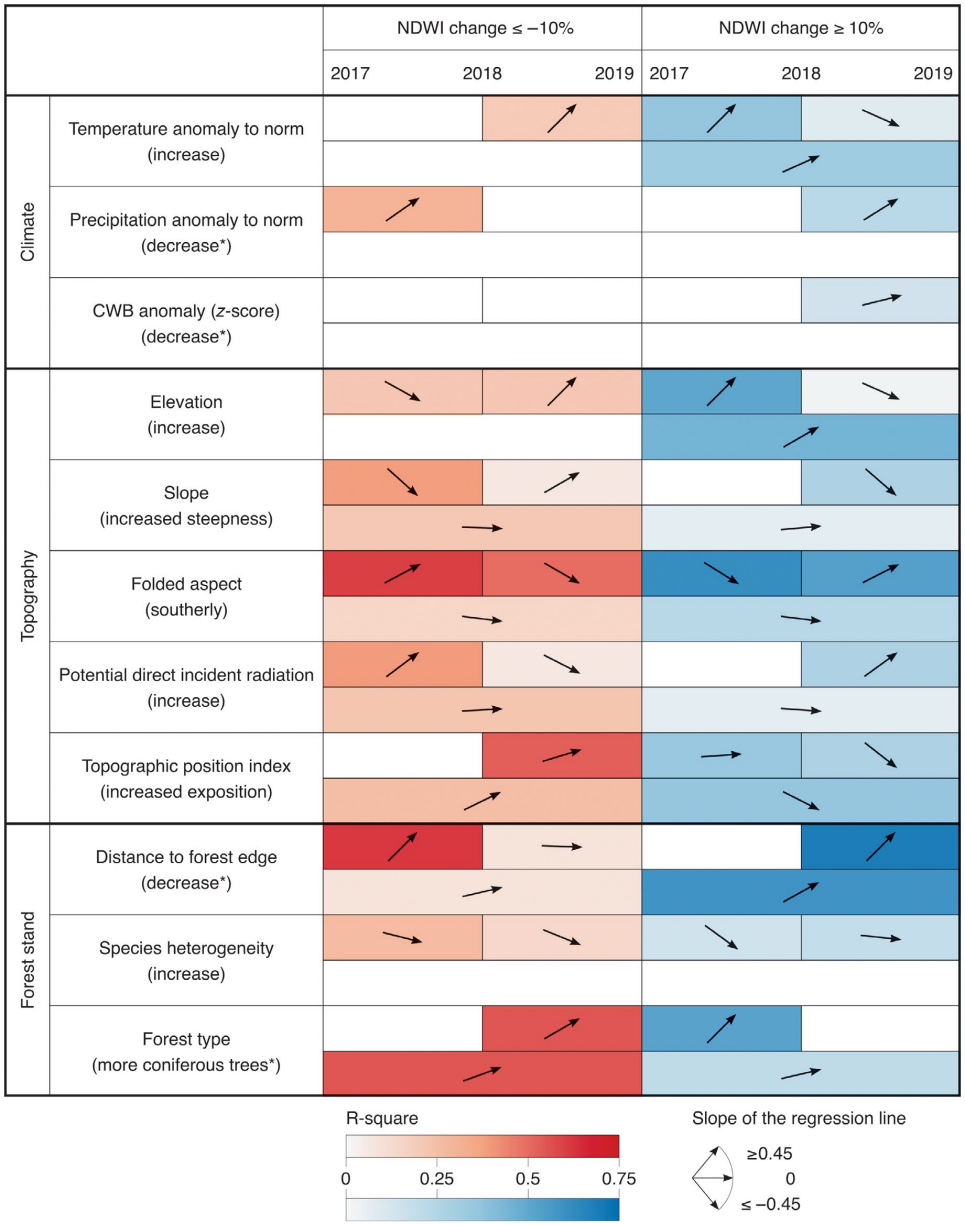
Linear models for environmental variables and positive or negative NDWI changes of ≥10% for 2017–2018 (resistance to the 2018 drought event), 2018–2019 (recovery), and 2017–2019 (resilience). The *R* ^2^ values of each relation are colored either in red (NDWI change ≤−10%) or blue (NDWI change ≥10%). The arrows indicate the slopes of the linear regressions (using the x‐axis with intervals from 1–10: changes in percentage affected pixels across the 10 intervals of each environmental variable). For the variables marked with a * the regression slopes are inverse to the range presented in Table 1 (max–min rather than min–max) to focus on drought characteristics (climate) or site characteristics where higher proportions of damaged forest were found (forest stand). The intensity of color represents the effect size as measured by the *R* ^2^ value of the regression. Fields of non‐significant relations (*p* > .05) are empty. Corresponding analyses with mean NDWI changes and with NDWI changes of ≥5% or ≥15% are presented in Figures S5–S7, respectively. These additional analyses yielded similar results to the ones presented here

Compared with the strong interannual difference in drought severity between the drought‐year 2018 and the pre‐ and post‐drought years 2017 and 2019, spatial variation in drought severity within the drought‐year across Switzerland was small. Thus, spatial variation in drought stress‐related climatic variables (2018 anomalies in temperature, precipitation, and CWB) only weakly affected forest drought response as measured by ≥10% NDWI changes (Figure 4). Nevertheless, the proportion of forested area with a ≥10% NDWI decrease from 2017 to 2018 (low resistance) was larger under high negative anomalies in precipitation (dry conditions), which was compensated by a larger area with positive recovery from 2018 to 2019 under high negative anomalies in mean precipitation and CWB z‐score. Conversely, the proportion of forested area with a ≥10% NDWI increase from 2017 to 2018 (over‐resistance) was larger under high positive anomalies in mean temperature (warm conditions), which was partly compensated by a larger area with negative recovery (≥10% decrease in NDWI) and a smaller area of positive recovery from 2018–2019, leaving a slightly larger area with ≥10% NDWI increase from 2017–2019 (over‐resilience) under high positive anomalies in mean temperature. This suggests that the temperature component of drought can actually be beneficial in areas with generally high humidity and low temperature (e.g., north‐facing slopes at high elevation).

### 3.3 Relation between further environmental variables and forest drought response

Compared with the above climatic variables, topographic variables and variables related to forest structure showed stronger linear relationships with the proportion of forested area with strong NDWI changes between years (Figure 4). The proportion of forested area with ≥10%NDWI decreases from 2017–2018, that is, low resistance, was strongly increasing from northern to southern aspects (Figure S2) and towards forest edges (for linear model despite a decreasing decline with larger distances as seen in Figure S3). The proportion of forested area with ≥10% NDWI increases from 2017–2018, that is, over‐resistance, increased along with elevation, was large on northern aspects and in forests with a large proportion of coniferous trees or higher species homogeneity. As mentioned in the previous section, these are likely areas with generally high humidity and low temperature and thus might have suffered less from the lowered precipitation and actually benefitted from the increased temperature component of the drought event.

The proportion of forested area with ≥10% NDWI decreases from 2018–2019, that is, negative recovery, was strongly increasing with the proportion of coniferous trees but also with elevation, steepness of slopes, and exposed positions. However, positive recovery was positively related to forest edge proximity, southern aspect, and high radiation, that is forest areas that were more negatively affected by the drought in the first place (low resistance, see above; in the next section recovery is analyzed separately for these areas with low resistance).

Regarding resilience, where no strong changes in NDWI from 2017 to 2019 indicate compensating resistance and recovery processes or generally small NDWI changes between adjacent years, strong changes (under‐ and over‐resilience) were comparatively weakly influenced by environmental variables. Where negative or positive ≥10% changes from 2017 to 2019 did occur, they were indicative of longer‐term negative effects of the drought event or overall positive effects of its temperature component, respectively. The proportion of forested area with strong NDWI decreases from 2017 to 2019, that is, under‐resilience or continuing negative effects of the drought year through the post‐drought year, was large in more exposed areas (i.e., forest located on a ridge or plateau; *R* ^2^ = .53) or in forests with a high proportion of coniferous trees. In contrast, the proportion of forested area with a strong NDWI increase from 2017 to 2019 (over‐resilience) was large at forest edges (*R* ^2^ = .59), in higher elevations, less exposed positions and again in forests with a high proportion of coniferous trees (Figure S4).

The results from the ≥10%‐change analyses presented in the previous three sections were further supported by the results from the direct NDWI‐change analyses (Figure S5) and from analyses using ≥5% (Figure S6) or ≥15% (Figure S7) changes in NDWI.

### 3.4 Recovery of forest areas after negative, neutral, or positive resistance

Out of the 4.29% of Swiss forests that showed ≥10% reductions in NDWI from 2017 to 2018, that is, low resistance to the 2018 drought event, 0.9% experienced a further decrease of NDWI from 2018 to 2019, 51.9% remained at the reduced level, and 47.2% recovered toward pre‐drought NDWI values.

**FIGURE 5:**
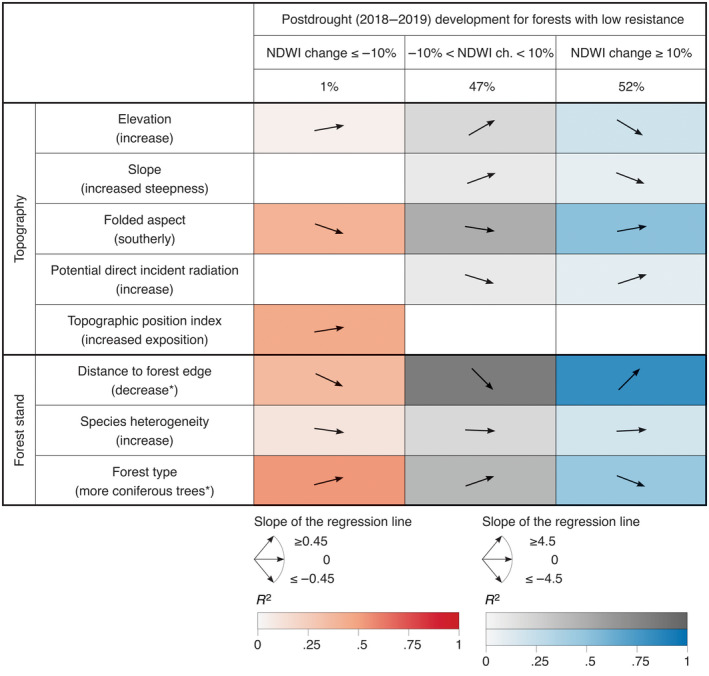
Forest recovery quantified as ≥10% change of NDWI from 2018 to 2019, for all forest areas with a negative NDWI change ≥10% from 2017 to 2018, that is, low resistance. The *R* ^2^ values of each relation are colored either in red for areas with further decreasing NDWI (negative NDWI change ≥10% from 2018 to 2019, i.e., negative resistance; 1% of cases), grey for areas with little further change in NDWI (change from 2018 to 2019 less than 10%; 47% of cases), or blue for areas recovering from low resistance (positive NDWI change ≥10% from 2018 to 2019, i.e., positive recovery; 52% of cases). The arrows indicate the slopes of the linear regressions between the proportion of forest areas with NDWI change and the affecting environmental variable (using the x‐axis with intervals from 1 to 10: changes in percentage affected pixels across the 10 intervals or each environmental variable; for the first column slopes are multiplied by 10 because here changes are small in relation to the percentage values). For the variables marked with a * the regression slopes are inverse to the range presented in Table 1 (max–min rather than min–max) to focus on the site characteristics where higher proportions of damaged forest were found. Fields of nonsignificant relations (*p* > .05) are empty

Forests with low drought resistance show highest proportions of compensating positive recovery (NDWI increase 2018–2019 ≥10%) near the forest edge, on south‐facing slopes, and for broadleaf trees (Figure 5). We generally found less recovering forest at higher elevation, on steeper slopes, at locations with lower potential incident radiation and in more homogenous forests, although these relationships had low *R* ^2^ values. The relationships were all inverted for forest areas that either experienced further NDWI decrease (NDWI decrease 2018–2019 ≥10%) or did not change by more than 10% in either direction from the lowered value after the drought event. Thus, areas with no or even negative recovery from 2018 to 2019 were found on north‐oriented slopes, at more exposed locations, further away from the forest edge, and in forests with a greater proportion of coniferous trees.

Results for forest “recovery” from 2018 to 2019 on pixels that were not affected by low resistance from 2017–2018 are shown in Figure S8 (NDWI changes <10% from 2017 to 2018) and Figure S9 (NDWI change ≥10% from 2017 to 2018, i.e., “over”‐resistance). Proportions of forest areas with either negative (2%) or positive (3%) recovery were small for forests with stable NDWI values from 2017 to 2018 (Figure S8). However, forest areas with ≥10% increased NDWI values from 2017 to 2018 often had negative recovery (23%), indicating compensation in the opposite direction from the typical one reported above of low resistance followed by high (= positive) recovery.

### 3.5 Combined effects of environmental drivers and their interactions

**FIGURE 6:**
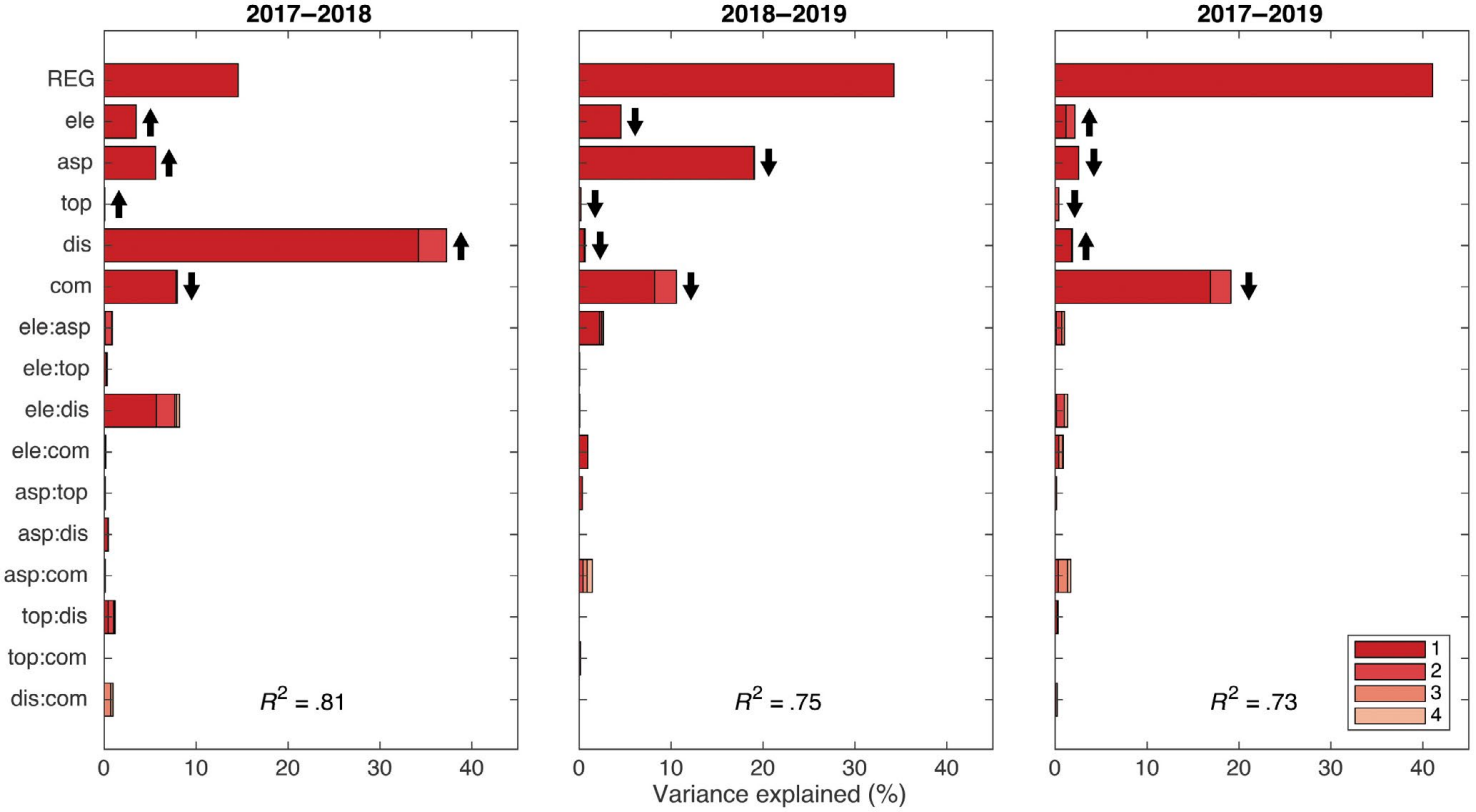
The variance explained by the mixed‐effects model combining the influence of different environmental drivers and their two‐way interactions on the proportion of forest pixels with a decrease in NDWI ≥10% during the drought (2017–2018, resistance), after the drought (2018–2019, “negative” recovery), and the full two‐year observation period (2017–2019, “under”‐resilience). The bars from top to bottom in each panel are the contributions to multiple *R* ^2^ values from biogeographic regions within Switzerland to elevation, aspect, topographic position, distance from forest edge, forest composition, and interactions of these (but not region). Below region, each bar is for the three‐level environmental driver or 9‐combination interaction fitted with two and four degrees of freedom, respectively (see Table S1, upper part). The shading within bars is used to indicate the contributions of 1‐degree of freedom contrasts: ele = contrast between lowland versus foothills and Alps, asp = north versus south, top = slopes versus valley‐bottoms and ridge‐tops, dis = forest edge versus non‐edge, and com = broadleaf trees versus mixed and coniferous trees. The strongest shading is for the contrasts or their interactions and the least shading for the deviation from the contrasts (Schmid et al., 2017). For interactions, the two intermediate shadings are for first‐named contrast interacting with deviation from second‐named contrast and vice versa; using lower case for contrasts and upper case for factors and deviations an interaction of two factors A:B expands to a:b (1 in inset legend) + A:b (2) + a:B (3) + A:B (4). The arrows behind the main effect contrasts point upward for a positive coefficient and downward for a negative coefficient

**FIGURE 7:**
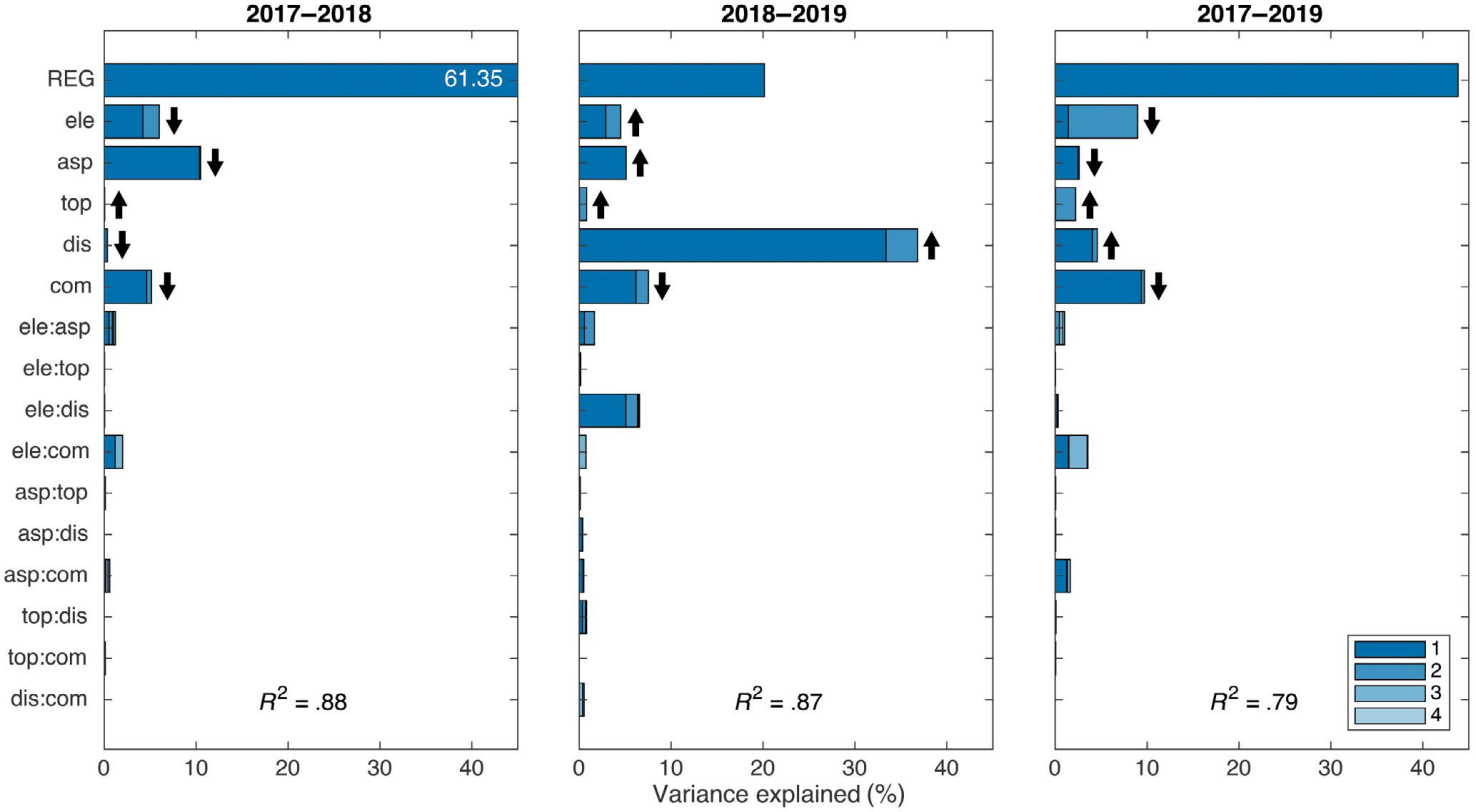
The variance explained by the mixed‐effects model combining the influence of different environmental drivers and their two‐way interactions on the proportion of forest pixels with an increase in NDWI ≥10% during the drought (2017–2018, “over”‐resistance), after the drought (2018–2019, recovery), and the full two‐year observation period (2017–2019, “over”‐resilience). The bars from top to bottom in each panel are the contributions to multiple R^2^ values from biogeographic regions within Switzerland to elevation, aspect, topographic position, distance from forest edge, forest composition, and interactions of these (but not region). Below region, each bar is for the three‐level environmental driver or nine‐combination interaction fitted with two and four degrees of freedom, respectively (see Table S1, lower part). The shading within bars is used to indicate the contributions of 1‐degree of freedom contrasts: ele = contrast between lowland versus foothills and Alps, asp = north versus south, top = slopes versus valley‐bottoms and ridge‐tops, dis = forest edge versus non‐edge, and com = broadleaf trees versus mixed and coniferous trees. The strongest shading is for the contrasts or their interactions and the least shading for the deviation from the contrasts (Schmid et al., 2017). For interactions the two intermediate shadings are for first‐named contrast interacting with deviation from second‐named contrast and vice versa; using lower case for contrasts and upper case for factors and deviations an interaction of two factors A:B expands to a:b (1 in inset legend) + A:b (2) + a:B (3) + A:B (4). The arrows behind the main effect contrasts point upward for a positive coefficient and downward for a negative coefficient

The analyses of the combined effects of the strongest environmental drivers from the separate regressions presented above confirmed the pattern found there, although here each variable was only represented with three intervals, here defined as categories, along its entire range. This method—which can be used for bias reduction in observational studies (Imbens & Rubin, 2015)—had the advantage that correlations between environmental drivers could be largely removed and thus the fitting sequence of drivers had little influence on their contribution to multiple R^2^ values, which we used to assess the contribution of single environmental drivers or their interactions to forest responses (Figures 6, 7). Thus, all tested environmental drivers (i.e., elevation, folded aspect, topographic position index, distance to the forest edge, and forest type), but not so much their two‐way interactions, were generally highly significant in linear mixed‐effects models with the factor region as random‐effects term (Table S1). The combined analyses had multiple *R* ^2^ values between .73 and .88, which is high compared with the large number of strata used in the analysis (see section Statistical analysis). A large contribution came from the random factor region (fitted first when calculating % SS), which was expected due to its higher degrees of freedom, but even after accounting for these regional differences, contributions from the tested environmental drivers were still large, together explaining typically around 40% (*R* ^2^ * 100—variance explained by regions in Figures 6, 7).

The combined analysis showed that the forest structure variables, *distance to forest edge* and *proportion of coniferous trees* were the most important drivers of forest responses to the 2018 drought; among the topographic variables, *aspect* and to a lesser degree *elevation* were important drivers. Using contrasts, we found that for *distance to forest edge* the largest differences in forest response was between forest edge (≤20 m) and forest interior. For *forest composition*, the largest differences were found between broadleaved and other forests, and for *aspect* between southern and other directions. However, *elevation* effects were only partly explained by the contrast lowland versus foothills and Alps, sometimes leaving larger differences between foothills and Alps (right panel in Figure 7). There were only three relatively strong interactions: forest edges in lowland areas (interaction “ele:dis”) had particularly low resistance (left panel in Figure 6) but high recovery (middle panel in Figure 7) and furthermore “over”‐resilience was particularly high in broadleaved forest at low elevations (interaction “ele:com” in Figure 7).

The similarity of results between the single‐variable regressions and the more complex combined analysis demonstrates that interdependencies between the drivers were relatively weak and thus the single‐variable regressions were little affected by confounding with the other drivers as potentially correlated “third variables.” Furthermore, the similarity of results also demonstrates that the conversion of environmental variables into 10‐ or 3‐interval variables did not mask the major trends these drivers had on resistance, recovery, and resilience. Finally, using mean NDWI changes per pixel instead of percentage pixels with NDWI changes ≥10% yielded qualitatively the same results (Table S2).

## 4 DISCUSSION

### 4.1 Resistance, recovery, and resilience of Swiss forests to the 2018 drought year

Recent studies indicate a substantial negative impact of the 2018 drought year on Swiss forests (Baltensweiler et al., 2020; Brun et al., 2020; Gharun et al., 2020; Rohner et al., 2021; Schuldt et al., 2020). Our satellite‐based assessment provides complementary insight and reveals varying and environmental gradient‐dependent forest drought impacts across entire Switzerland. The observed strong decrease in NDWI in the northern and western regions of Switzerland (along the Jura mountains) for 2017–2018 and 2017–2019 corroborates results from local field assessments (BAFU, 2019). The lowered resistance in the North was expected due to low CWB z‐scores indicating the strong drought extent in the North. Based on a ≥10% reduction in NDWI from the pre‐drought year 2017 to the post‐drought year 2019 (here termed “under”‐resilience), we estimated that 3.54% of the Swiss forest was not resilient to the drought event of 2018, at least not in the short term of one year following the event. Although not directly comparable due to different categorical approaches, this number is within the range of field assessments estimating a substantial crown defoliation (>60%) for Norway spruce (5.9%) and European beech (2.8%) or 4.7% and 1.2% of trees dying after the drought event, respectively (BAFU, 2019). This reinforces the suitability of satellite‐based assessments exploiting the NDWI at a 10 × 10 m pixel scale as proxy for forest drought damage. Nevertheless, continuous NDWI changes and defoliation rates would be needed to establish the direct relationship of the two measurements.

Reported percentages of affected areas are the highest on record for Switzerland and they are even considerably larger than the second highest mortality rates recorded in 1994. Comparable or even higher defoliation rates for 2018 and 2019 were observed in Germany for Norway spruce (4.2%) and European beech (6.2%), respectively, whereas mortality rates in Germany were lower for both species (BMEL, 2020). During the drought, surprisingly, we also observed increased NDWI values for 3.76% of the forested area. One possibility is that this was due to overlapping timelines of tree recovery from previous drought events. The recovery from drought in forests might take 2 to 4 years (Anderegg et al., 2015) and thus forests with a high resistance to the 2018 drought event might actually represent late recovery from the 2015 drought. Another possibility is that the high‐temperature component of the drought event 2018 had a beneficial effect in humid and cold areas (e.g., north‐facing slopes at high elevations), where water availability was still sufficient and higher temperature led to increased growth. We speculate that NDWI decreases ≥10% from 2018 to 2019 (negative recovery) at low elevations indicated continued negative effects of the 2018 drought, whereas at high elevations they were likely compensatory responses to NDWI increases ≥10% from 2017 to 2018 (negative resistance). Longer time series would be required to distinguish these two types of trajectories and to see if delayed recovery may have occurred in lowland areas.

Areas with NDWI increases ≥10% from 2018 to 2019 (positive recovery) where often the same as those affected by NDWI decreases ≥10% from 2017 to 2018 (low resistance), indicating compensatory recovery after losses due to the drought event. This was particularly the case on south‐facing slopes and near forest edges as further discussed in the next section. In fact, 52% of the forest damaged in 2018 recovered in 2019 (Figure 5). The compensatory recovery may in part be explained by an increased water availability in 2019, due to a regionally wet winter and spring as well as sufficient precipitation in the summer 2019 (MeteoSchweiz, 2020), and tree mortality in 2018 thus reduced competition as found in previous studies (Cavin et al., 2013; Rohner et al., 2021; Vitasse et al., 2019).

Surprisingly, highest proportions of strong NDWI decreases from 2017–2019 (“under”‐resilience) were found in the Northwest and Southeast, where the drought (i.e., CWB z‐score) was not as prominent as in other regions of Switzerland. In the Northwest this could have been due to additional heavy bark beetle outbreaks (BAFU, 2019) that decreased the recovery potential of Norway spruce and caused high tree mortality rates. Indeed, we found that forest composition was one of the strongest moderators of forest drought responses, with forests containing conifers showing significantly stronger decreases in NDWI from 2018 to 2019 and 2017 to 2019 than mixed forests or forests dominated by broadleaf trees. Additional stressors thus can diminish the natural recovery potential of forests (Dobbertin et al., 2007; Lévesque et al., 2013; Miralles et al., 2019).

For the validation of our threshold‐based approach (i.e., ≥10% NDWI change), we compared our results with visual inspections of high‐resolution satellite imagery (Figure 2), resulting in an overall accuracy over 83%. The producer's accuracy for forests not affected by drought was almost 99%, whereas the producer's accuracy for damaged forest was 18%. This indicates that the applied threshold only assessed severe forest damages; to include less severe damages, the threshold could be lowered. The user's accuracy was 84% and 77% for intact forest and damaged forest, respectively. Depending on the aim of further analysis the applied threshold could be adapted. However, when we used other cutoff values of ≥5% or ≥15% NDWI change (Figures S6 and S7 respectively) or even mean NDWI changes directly (Figure S5 and Table S2), we obtained similar results as with the ≥10% cutoff.

### 4.2 Effect of environmental drivers on forests in response to the 2018 drought

Our two types of analyses consistently showed that forest response to drought was strongly related to topographic site characteristics and forest stand structure, whereas climatic variables and interactions between drivers had comparatively small (yet still significant) effects. The same effects of drivers could be observed if they were stratified each into 10 or only 3 intervals. Because in the second case the drivers could be combined into a single analysis where they became more orthogonal to each other, we could show that their contributions to forest drought responses were largely independent of each other and, after correcting for differences between regions (used as random‐effects term in our mixed‐model analyses), they jointly explained a large proportion of variance in forest drought response, reducing unexplained variation to only around 20% (see Figures 6, 7). Thus, given that the results of the combined analysis were consistent with those of the separate analyses and given the weak interaction effects in the combined analysis, we focus the remaining discussion on the results from the separate analyses for each driver.

During the drought event, less resistant forests (i.e., higher proportions of strong NDWI decrease from 2017 to 2018) were more often observed on south‐ than on north‐facing slopes, whereas this changed during recovery from 2018 to 2019 and over the whole period from 2017 to 2019, when the proportion of damaged forest was higher on north‐oriented slopes. This suggests that regarding recovery and resilience, remarkably trees of all forest types were better adapted to drought on drier and warmer slopes, which has been reported previously for mixed conifer forests (Guarín & Taylor, 2005).

Furthermore, forests in topographically exposed locations (i.e., on ridges) had both lower resistance and resilience than forests in valleys, as hypothesized. As mentioned in the first paragraph of this section, these drivers were only weakly correlated with (high) elevation and had only weak interactive effects with elevation, which in contrast had rather positive effects on resistance and resilience (see Figure 4), presumably due to positive effects of temperature. Wind and radiation affect lager parts of the forest canopy by increasing evapotranspiration when it is located in exposed terrain, in comparison with sheltered and shaded forest stands. Another explanation is the higher buffering of soil water availability in sheltered downhill positions, whereas ridge positions have lower buffering of soil water availability and thus may cause higher water stress (Hawthorne & Miniat, 2018). This buffering has several components, such as closer proximity to groundwater at less exposed positions (Western et al., 2001) and input‐independent higher recession of soil moisture at exposed locations (Hawthorne & Miniat, 2018), indicating the dominance of topography over soil properties as a driver of forest responses to droughts (Yeakley et al., 1998). Also trees in ridge locations are reported to have generally more extensive root systems (Tauc et al., 2020) and may thus be genetically better pre‐adapted to drier conditions than trees growing in other locations (de Kort et al., 2020). Nevertheless, our analysis showed that these potential pre‐adaptations were apparently not sufficient to protect those trees against the water stress experienced in 2018.

At higher elevation we found smaller proportions of forested area with severe NDWI decrease during the 2018 drought confirming findings from previous studies (Lloret et al., 2007; Lobo & Maisongrande, 2006; Rita et al., 2020). Simultaneously, strong NDWI increases were prominent in the alpine regions during the drought year, which were historically energy‐ rather than water‐limited (Schurman et al., 2019). With higher temperature there are more frost‐free days (Jolly et al., 2005), growth conditions closer to the optimum (Klesse et al., 2018), and a considerably longer growing season (Schurman et al., 2019). According to one study, these improved growing conditions resulted in increased net CO_2_ uptake and increased gross primary production in 2018 compared with 2017 in some regions (Gharun et al., 2020). Thus, the high‐temperature component of the 2018 drought event might have been beneficial in humid, cold alpine areas and have outweighed the negative effects of precipitation deficits, leading to increased growth, increased canopy water content, and thus higher NDWI values (Scherrer & Körner, 2011). Positive impacts of warmer temperatures on energy‐limited forest ecosystems have been found often (Dawes et al., 2011; Seddon et al., 2016). However, as discussed above, the over‐resistance to the drought event in 2018 was often followed by a negative recovery in 2019 at these locations, suggesting a relaxation effect from the growth spurt under conditions less limiting due to increased temperatures. Furthermore, water availability in alpine areas is highly influenced by the snowpack which was not included in the present analysis. As reported in a previous study (Gazol et al., 2017), we found increasing drought resistance on steeper slopes. Forest stands on steep slopes are perhaps more used to reduced water availability due to faster run off.

Among the drivers related to forest stand structure, forest edges were clearly more exposed to drought damage as reflected in their low resistance values to the 2018 drought. This confirmed our hypothesis and has also been observed for other drought events (Buras et al., 2018; Laurance & Williamson, 2001). Reasons for the higher vulnerability to water stress at forest edges might be the less favorable micro‐climatic conditions such as higher temperatures not just at the top of canopy but also down to the soil surface (Buras et al., 2018; Chen et al., 1993) as well as lower relative humidity (Von Arx et al., 2012) that cause higher evaporation and thus accelerate soil water stress. In contrast, further inward in a forest stand the canopy provides shade that leads to reduced temperatures and lower evapotranspirative demand (Grote et al., 2016) compared with forest edges. In general, competition for water and light should be less strong at forest edges, which might explain the higher recovery rates, but this may not have compensated the other microclimatic effects during the drought. Higher post‐drought recovery rates have also been observed in tropical forests (Schwartz et al., 2019).

Whereas the linear model fit between drought resistance and forest composition was nonsignificant, forest composition was the most important driver of resilience in our study. As hypothesized, forests with a high proportion of coniferous trees were more damaged by the drought than forests with another composition in the longer term from 2017–2019. This finding corroborates earlier suggestions from field‐based studies (Arend et al., 2021; Lévesque et al., 2013; Vitasse et al., 2019) and may be explained by the shallower rooting depths of Norway spruce, while European beech has a deep root system that reaches water in deeper soil layers (Bréda et al., 2006; Brinkmann et al., 2019). Additionally, bark beetle outbreaks affect spruce and can weaken recovery after a drought event (Biedermann et al., 2019). In contrast to our findings, a drought in Poland in 2015 (Nasiłowska et al., 2019) affected coniferous and mixed forests less than deciduous forests. This different finding is most likely related to the timing of the drought event. The drought in 2015 in Poland started earlier and influenced leaf and needle flush producing visible damages. For conifers, however, this effect might be diluted by the presence of older, less vulnerable needles. Additionally, while European beech is the most prominent deciduous species in Switzerland, the study site of Nasiłowska et al. (2019) is dominated by pedunculated oak (*Quercus robur*), a more drought resistant species than European beech (Scharnweber et al., 2011), additionally indicating the importance of timing in drought events (D’Orangeville et al., 2018).

Low resistance is usually followed by high recovery (Gazol et al., 2017), as we observed for example for south‐facing slopes and near forest edges. The net effect is then a resilient drought response. However, resilience can also be achieved by generally small changes in response to extreme events, that is, small negative combined with small positive changes. This has been observed in particular for diverse forests (Anderegg et al., 2018; Gazol & Camarero, 2016; Jucker et al., 2014; Pardos et al., 2021), for example, through facilitation between species (Pretzsch et al., 2013). Indeed, also in our study, more heterogeneous forests also showed this type of drought response when compared with more homogeneous forests. This confirms our hypothesis of diverse forest stands being less vulnerable to drought, presumably due to niche partitioning reducing interspecific competition.

In contrast to our hypothesis, most linear fits between proportions of forest area with strong NDWI changes and climatic variables (i.e., mean temperature anomaly, total precipitation anomaly, and CWB z‐score) were largely not significant (*p* > .05). We did observe more damaged forest during the drought under drier conditions, but the combination with temperature showed no relations. The lack of associations between forest drought responses and climatic variables in our study may be due to the fact that the drought year 2018 was so extreme that differences in “extremeness” did not matter much or perhaps to the different spatial resolution of the input data (Allen et al., 2010, 2015; Brun et al., 2020). The coarse spatial resolution of the weather data suppresses important local variation especially in highly varying topography (Macek et al., 2019; Potter et al., 2013; Slavich et al., 2014), which is especially the case for Switzerland where most forest is found on hillslopes rather than on flat areas.

### 4.3 Trajectories of drought‐induced forest damage

Although the aggregation of data into strata makes it more difficult to follow trajectories of individual pixels, we could do so in a more general way by adding to the overall analysis a conditional analysis in which we analyzed recovery in regard of the 2018 drought for different resistance scenarios in 2018 (negative, neutral or positive NDWI changes from 2017–2018). This first post‐drought year is indicative for both positive and negative drought legacy effects, which have been found to affect up to three subsequent years (Anderegg et al., 2015). A large proportion of forest pixels with low resistance (see Figure 5) did recover toward pre‐drought values of 2017 in 2019 (52%), while the remainder either stayed at low levels (47%) or reduced further (1%) indicating negative legacy effects. Overall, we found the same pattern of effects of drivers on the drought response of forest pixels with low resistance as for all pixel analyses discussed in the previous section. In summary for the pixels with low resistance to the drought event in 2018, we observed larger recovery in lower elevations, on flatter terrain, on southern slopes, and on sites with a higher potential direct incident radiation, as well as at forest edges and in stands with higher tree species heterogeneity. Furthermore, forests with a high proportion of deciduous trees recovered better than forests with abundant coniferous trees, suggesting functional group‐specific adaptation strategies. Although we already discussed the potential explanation for the effects observed in the overall analysis, we reiterate the positive drought legacy effects like they have been observed by Kannenberg et al. (2019) such as increased water (Cavin et al., 2013; Rohner et al., 2021; Vitasse et al., 2019) and nutrient availability (Gessler et al., 2017; Van der Molen et al., 2011) for a patch of damaged trees in this conditional analysis due to mortality and therefore reduced competition within this patch.

### 4.4 Reliability of this study

Our analysis of satellite‐based data provides a consistent and ground‐comparable way to assess drought‐induced forest changes over large areas (Gazol et al., 2018). Nevertheless, several limitations and assumptions must be considered when interpreting the results. Satellite‐based monitoring of drought only allows to evaluate state and changes of the upper tree crown (Damm et al., 2020), while possible damages happening lower in the canopy or even at stem level are not visible. Nevertheless, defoliation leads to translucency of the canopy crown and reflectance from the understory adds (unwanted) variation to the spectral signal (Barry et al., 2008). Additionally, although the used 10 × 10 m pixel size comes close to the diameter of individual canopy trees, pixels seldom represent individual trees measured in field surveys (Vicente‐Serrano et al., 2013; Zheng et al., 2021).

Clouds and cloud shadows affect measured radiances and sometimes hinder a robust retrieval of surface information. While clouds can be well masked, the identification of cloud shadows is more complicated and the separation between cloud and topographic shadow is not always possible. Our cloud‐masking and gap‐filling approach was conservative, resulting in a consistent exclusion of cloud shade at the cost of the additional exclusion of a small proportion of forested areas shaded by topography.

The used NDWI is sensitive to canopy water content, as demonstrated by our sensitivity analysis. However, observed NDWI changes do not only represent drought effects on canopy water content but may also reflect dynamics in canopy leaf area (Gao, 1996). It must be noted that leaf area can also be a result of land cover change (harvest) and damages caused by storms, pathogens, and bark beetle (*Ips typographus* L.) outbreaks (Hotovy & Jenicek, 2020; Olthof et al., 2003; Wood et al., 2018). Because we did not disentangle the contribution of these individual factors on observed NDWI changes, we cannot claim causality between drought and forest damages in all cases.

For our study, we took 2017 as a reference year, although summer temperatures were already high compared with a longer reference period (MeteoSchweiz, 2017). The contribution of possible pre‐stress could have increased vulnerability and caused legacy effects from earlier events (e.g., the drought in 2015) for which trees might on average take 2 to 4 years to recover (Anderegg et al., 2015). Such effects cannot be separated with only one base year. A longer time series including different sensor systems such as Landsat imagery at 30 m spatial resolution would be needed to increase the reference period and account for potential legacy effects. Furthermore, we only looked at the first year after drought and thus could not assess potential longer‐term recovery or decline, including additional stressful or beneficial effects during later years. There is a possibility that not only growth responses but also canopy water status recovery might have continued beyond 2019. If the time span between drought events becomes shorter than the recovery phase of forest ecosystems, permanent damage is to be expected (Schwalm et al., 2017). We plan to apply the methods introduced here for the analysis of further years to potentially detect long‐term legacy effects of the 2018 drought event.

We applied a 20 m inward buffer on the forest mask to avoid mixed pixels along the forest border. This filtering probably resulted in an underestimation of the total forested area affected by drought because drought tends to cause greater damage at forest edges. The applied forest mask is from 2020 and land‐use changes in forest areas are thus included in the analysis. The only solution to this problem would be to generate a forest mask from the spectral imagery for each of the years 2017, 2018, and 2019 and restrict the analysis to the areas classified as forest in all years. Additionally, the forest mask does include smaller clearings (e.g., in the north‐east and south‐west of the forest area in Figure 2d,e,f) and forest trails resulting in mixed signals that biased the NDWI maps. Particularly the drought response of grassland as depicted by NDWI changes is more extreme than the response from forests; and mixed spectral signals result in reduced resistance and resilience values and higher recovery values. Furthermore, mixed signals due to understory could not be erased. The understory might have been less affected due to partial shade and lower temperatures (Grote et al., 2016) and thus dampened the measured decrease in NDWI or led to an underestimation of drought damage in 2018, since shallow‐rooted understory may have experienced water stress earlier than deep‐rooted trees. Further, mixed pixels may cause an overestimation of forest recovery due to beneficial effects of resource reallocation from stressed trees to other plants. Non‐congruent spatial overlap of NDWI maps with spatial environmental variables from different data sources may reduce correlations between these variables and forest drought response. The coarser spatial resolution of precipitation and temperature data, for example, might have dampened a possibly stronger correlation with forest drought response. Thus, data at a finer spatial scale would be needed. Locally more precise data could be obtained from measurement stations, with the disadvantage of spatial nonconsistency. Alternatively, weather data could be improved by integrating a DEM at finer spatial resolution (Fang et al., 2013; Hutengs & Vohland, 2016). Finally, the list of environmental variables used in this study is not complete and thus cannot fully explain drought‐induced impacts on forest ecosystems. We did, for example, not consider soil properties such as soil water holding capacity that are directly related to plant growth (Piedallu et al., 2011) and can thus also affect and partly explain forests’ response to drought events.

Finally, we used an indirect analysis approach in the way of aggregating pixels, which had NDWI responses passing a threshold of 10%. We have also analyzed the direct values of NDWI change (Figure S5 and Table S2), that is, the mean resistance, recovery, and resilience values, which showed equal responses to the analyses with the proportions of severely affected forest areas. This justifies our indirect analysis approach. Furthermore, different threshold values (Figures S6 and S7) revealed again similar results but with lower R^2^ values, s indicating that our chosen threshold of 10% differentiated well between damaged and non‐damages trees.

## 5 CONCLUSIONS

We conclude that satellite‐derived measurements of the NDWI are applicable to assess the impact of extreme events such as droughts on the structure and functioning of forest ecosystems across large contiguous areas such as entire Switzerland. We set to further elucidate drought responses in temperate forests and increase our understanding of how such ecosystems respond to this increasingly more frequent stressor. We found that forest stand characteristics such as distance to forest edge and functional group composition (deciduous broad‐leaved vs. needle‐bearing coniferous species) strongly affected drought response. This suggests reducing edge length per area and conifer component as potential management strategies to avoid negative effects of an increasing frequency of drought events. We also found that forests on southern slopes were more vulnerable to drought than forests on norther slopes, suggesting that drought events may push forests in already exposed conditions beyond their “adaptive” limits, possibly requiring replanting with more drought resistant genotypes or species. The effects of individual environmental variables on forest drought response were additive (only weak interactions) and valid across regional differences in Switzerland. Extrapolation beyond Switzerland would have to be carefully tested for different forest types and site characteristics to see if our approach is applicable to other temperate biomes where drought events in combination with higher temperatures increase in frequency and become more important in damage assessments.

Further research should expand the analysis of continuous time series rather than three month composites from successive years for a better understanding of the dynamics of forest response to severe water stress. This would also enable an assessment of the optimal timing of satellite‐based drought surveys considering accumulated damage and evolving stress symptoms. Additionally, a better understanding of underlying mechanisms influencing drought will be needed to develop mitigation strategies against future drought events.

## CONFLICT OF INTEREST

The authors declare no conflict of interest.

## AUTHOR CONTRIBUTIONS

JS, AD, and MJS designed the study. JS performed the data collection and analysis and wrote the manuscript together with inputs from AD, MJS, and BS.

## Supporting information

Supplementary Material

## Data Availability

The data that support the findings of this study are openly available in Dryad at https://doi.org/10.5061/dryad.bk3j9kdd8.
