# Supplementary material for: Satellite data reveal differential responses of Swiss forests to unprecedented 2018 drought
Source: Glob Chang Biol. 2022 Mar 7;28(9):2956–78. doi: 10.1111/gcb.16136 (PMC9310759; doi:10.1111/gcb.16136)
Supplement: Supplementary file 1 — Supplementary Material [file GCB-28-2956-s001.docx]

Satellite data reveal differential responses of Swiss forests to unprecedented 2018 drought

Joan Sturm, Maria J. Santos, Bernhard Schmid, and Alexander Damm

**Supplementary Online Information**


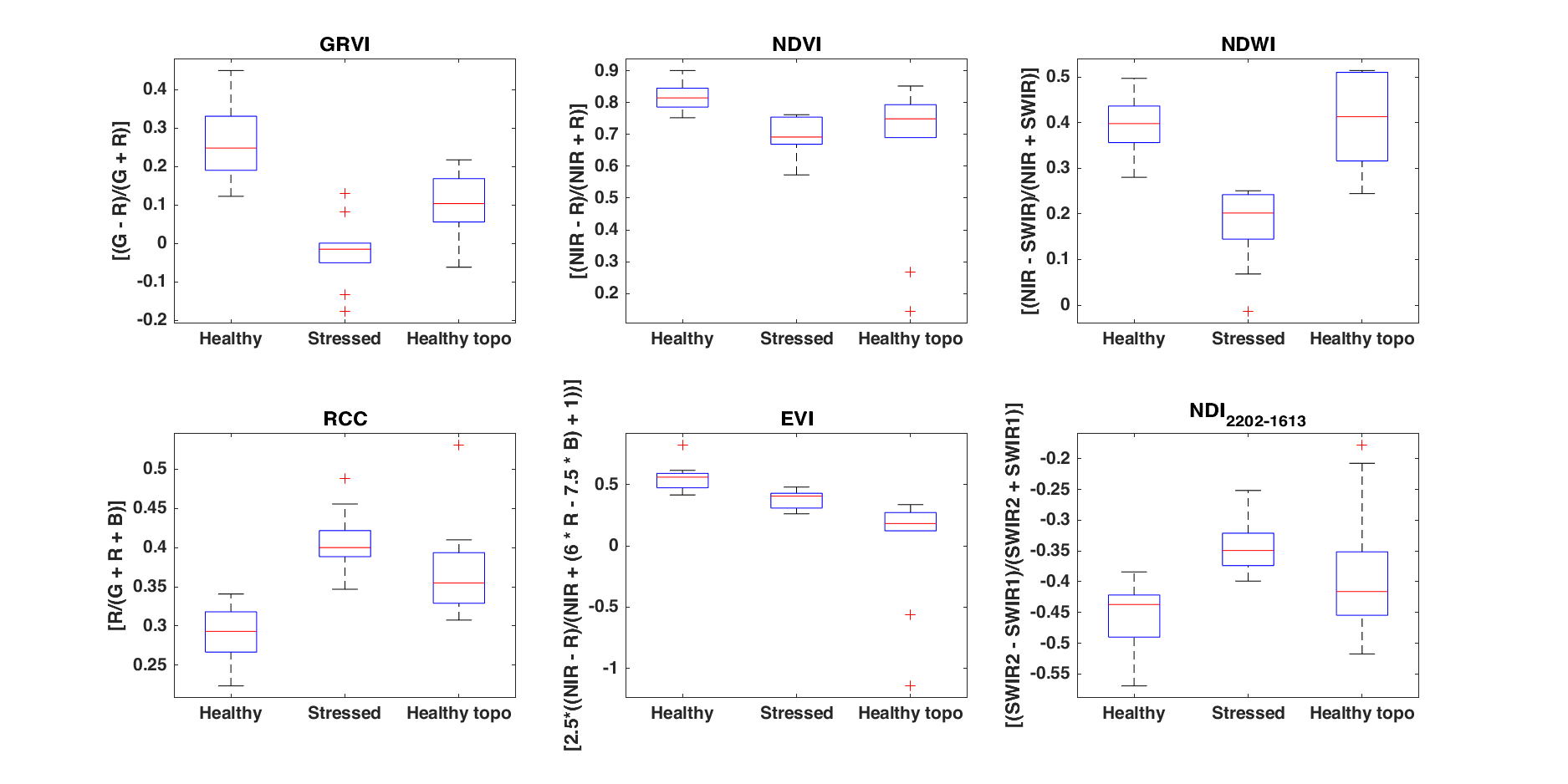


Figure S1: Sensitivity analysis of vegetation indices used to detect plant water stress applied to (i) forest appearing healthy on flat terrain, (ii) forest visibly damaged (leaf discoloration) on flat terrain, and (iii) forest appearing healthy on steeper slopes. On the left are indices using only bands from the visible spectrum including the green-red vegetation index (GRVI) (Tucker, 1979), and the red chromatic coordinates (RCC) (Gillespie et al., 1987), in the middle we have indices using both the visible and near infrared wavelength regions including the normalized difference vegetation index (NDVI) (Tucker, 1979), and the enhanced vegetation index (EVI) (Huete et al., 2002), and on the right are indices using longer wavelengths including the normalized difference water index (NDWI) (Gao, 1996), and a normalized difference index of Sentinel-2’s short wave infrared bands (NDI).


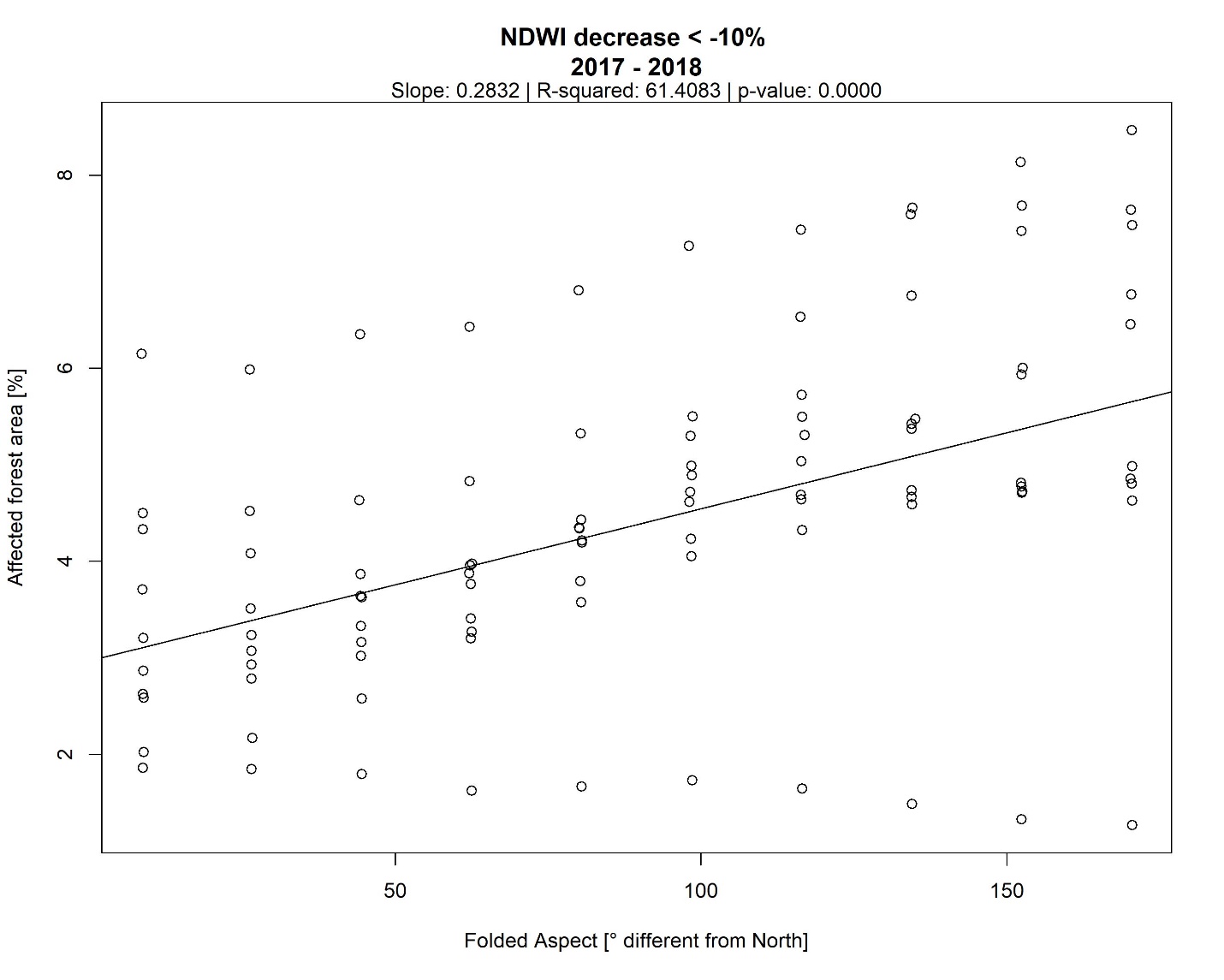


Figure S2: Scatterplot for the relation between the site characteristic folded aspect [° different from North] and the proportion of forest area with a NDWI change ≤ -10% from 2017–2018. The range of 180° has been divided into ten intervals and for each interval the percentage of heavily affected forest pixels has been calculated for 10 biogeographic subregions in Switzerland (circles). The fitted linear model used values from 1 to 10 for the ten classes along the x-axis and has been weighted by the absolute number of forest pixels in each interval and corrected for the subregions to generate the regression line and calculate its coefficient of determination (R-squared).


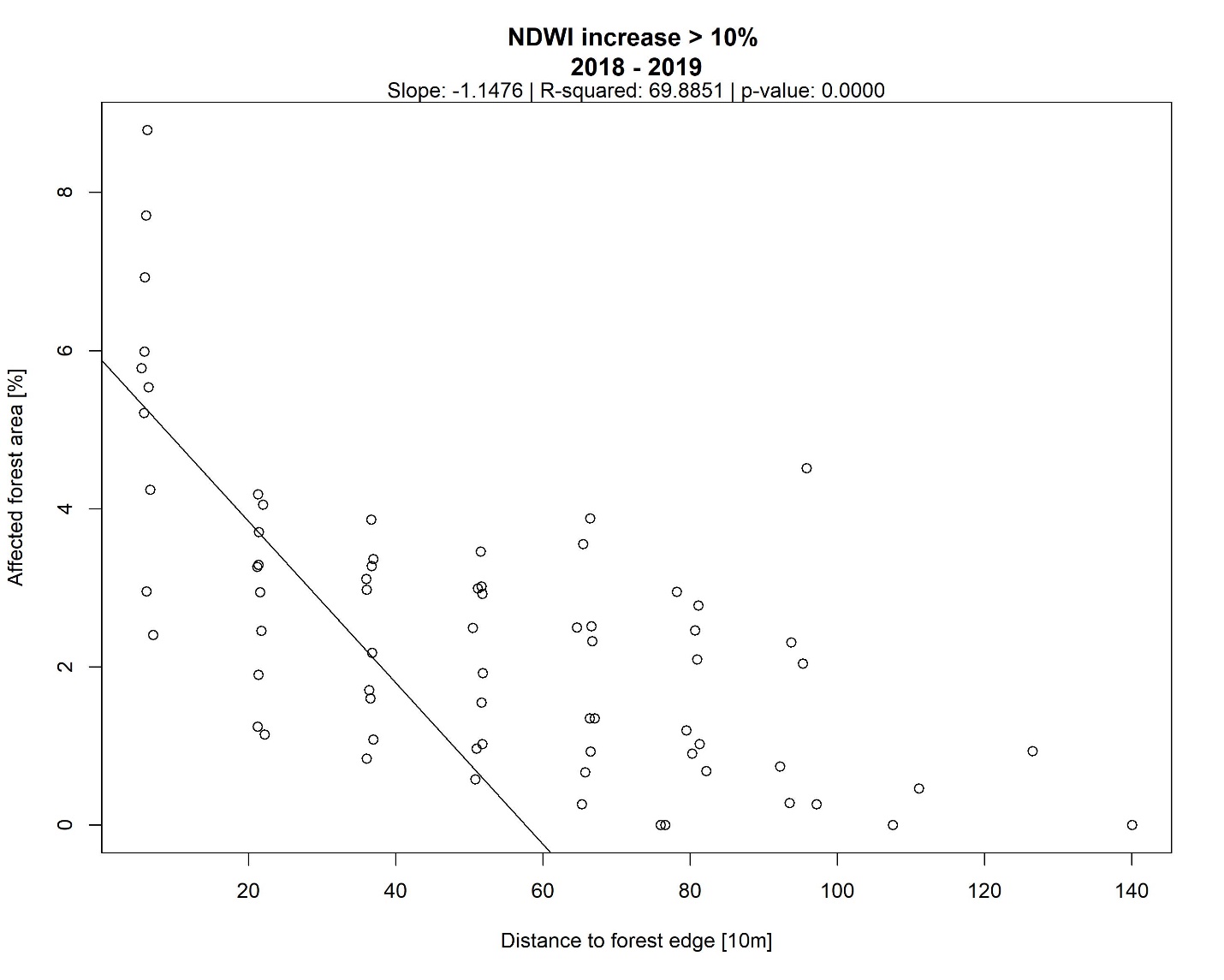


Figure S3: Scatterplot for the relation between the stand characteristic distance to forest edge [10 m] and the proportion of forest area with a NDWI change ≥ 10% from 2018–2019. The range of 1400 m has been divided into ten intervals and for each interval the percentage of heavily affected forest pixels has been calculated for 10 biogeographic subregions in Switzerland (circles). The fitted linear model used values from 1 to 10 for the ten classes along the x-axis and has been weighted by the absolute number of forest pixels in each interval and corrected for the subregions to generate the regression line and calculate its determination coefficient (R-squared). Note that in this case a better fit would be obtained with transformed distance (e.g. close (< 10 m) vs. far or log distance; however, for consistency with other regressions, we kept the linear scale.


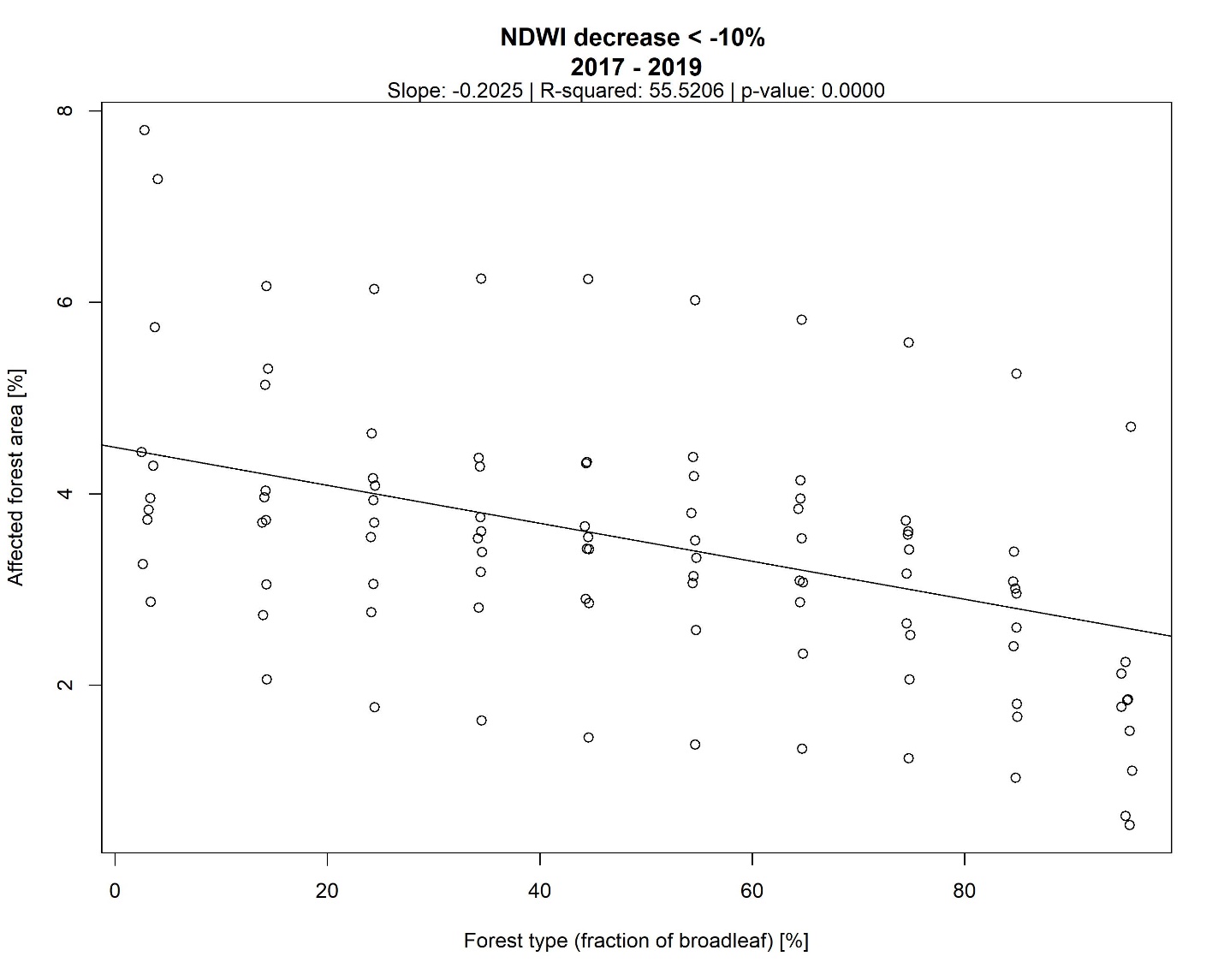


Figure S4: Scatterplot for the relation between the stand characteristic forest type expressed as the fraction of broadleaf trees [%] and the proportion of forest area with a NDWI change ≤ –10% from 2017–2019. The range of 100% has been divided into ten intervals and for each interval the percentage of heavily affected forest pixels has been calculated for 10 biogeographic subregions in Switzerland (circles). The fitted linear model used values from 1 to 10 for the ten classes along the x-axis and has been weighted by the absolute number of forest pixels in each interval and corrected for the subregions to generate the regression line and calculate its determination coefficient (R-squared).


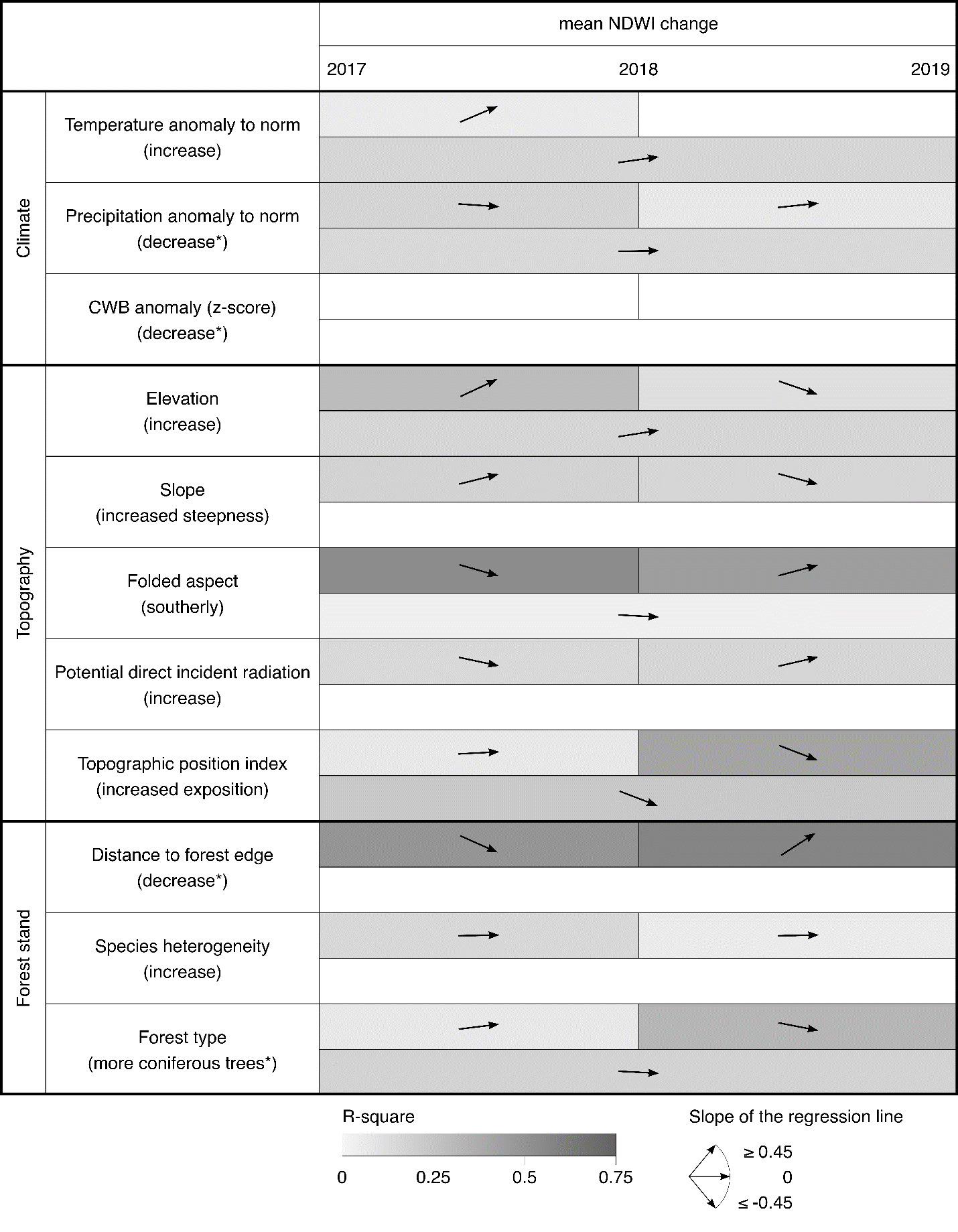


Figure S5: Linear models for environmental variables and the mean NDWI change for 2017–2018 (resistance to the 2018 drought event), 2018–2019 (recovery), and 2017–2019 (resilience). The R^2^ values of each relation are colored in grey. The arrows indicate the slopes of the linear regressions (using the x-axis with intervals from 1–10: changes in mean resistance, recovery, and resilience values across the 10 intervals of each environmental variable). For the variables marked with a * the regression slopes are inversely to the range presented in Table 1 (max–min rather than min–max)­ to focus on drought characteristics (climate) or site characteristics where higher proportions of damaged forest were found (forest stand). The intensity of color represents the effect size as measured by the R^2^ value of the regression. Fields of non-significant relations (*p* > 0.05) are empty.


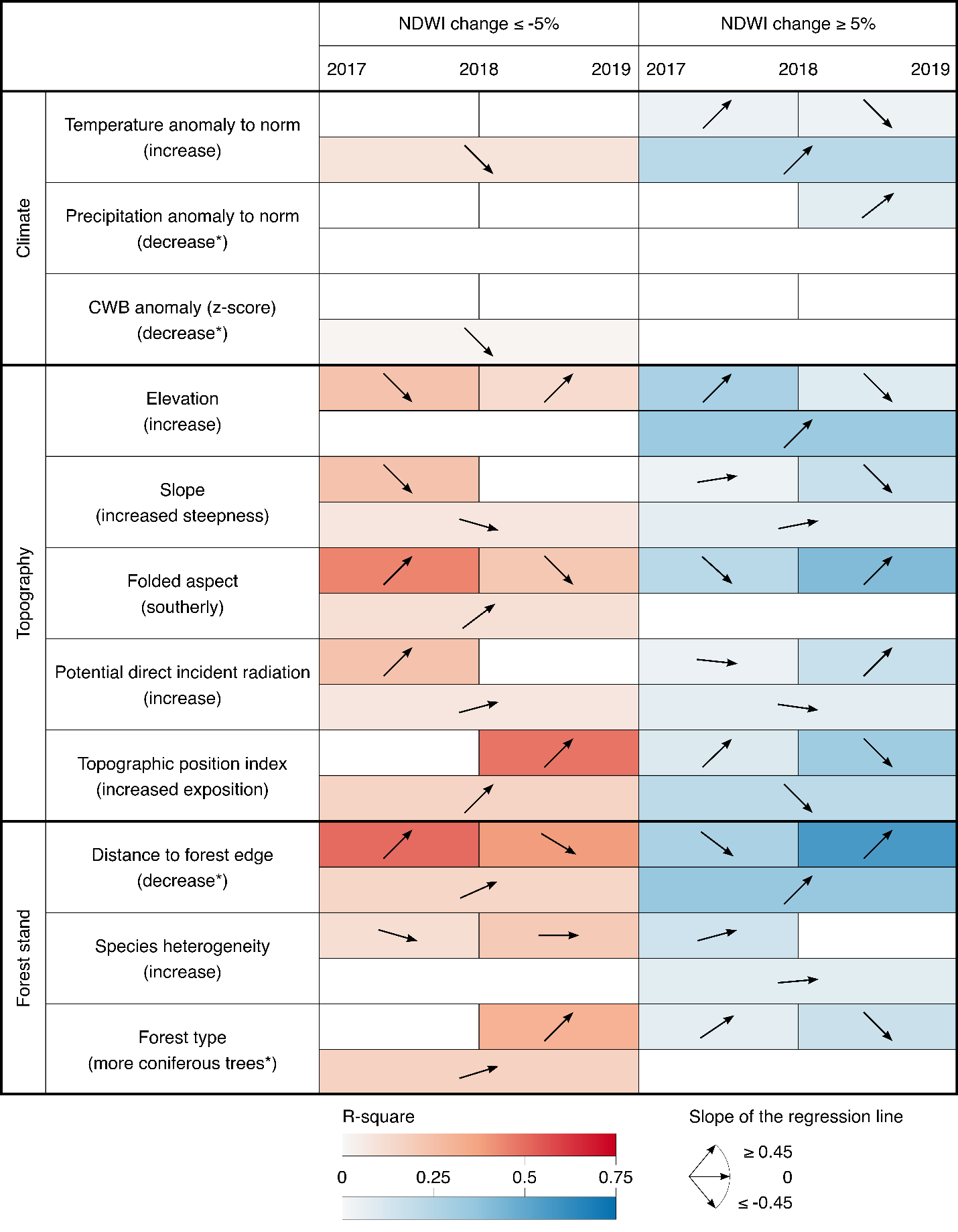


Figure S6: Linear models for environmental variables and positive or negative NDWI changes of ≥ 5% for 2017–2018 (resistance to the 2018 drought event), 2018–2019 (recovery), and 2017–2019 (resilience). The R^2^ values of each relation are colored either in red (NDWI change ≤ -5%) or blue (NDWI change ≥ 5%). The arrows indicate the slopes of the linear regressions (using the x-axis with intervals from 1–10: changes in percentage affected pixels across the 10 intervals of each environmental variable). For the variables marked with a * the regression slopes are inverse to the range presented in Table 1 (max–min rather than min–max)­ to focus on drought characteristics (climate) or site characteristics where higher proportions of damaged forest were found (forest stand). The intensity of color represents the effect size as measured by the R^2^ value of the regression. Fields of non-significant relations (*p* > 0.05) are empty.


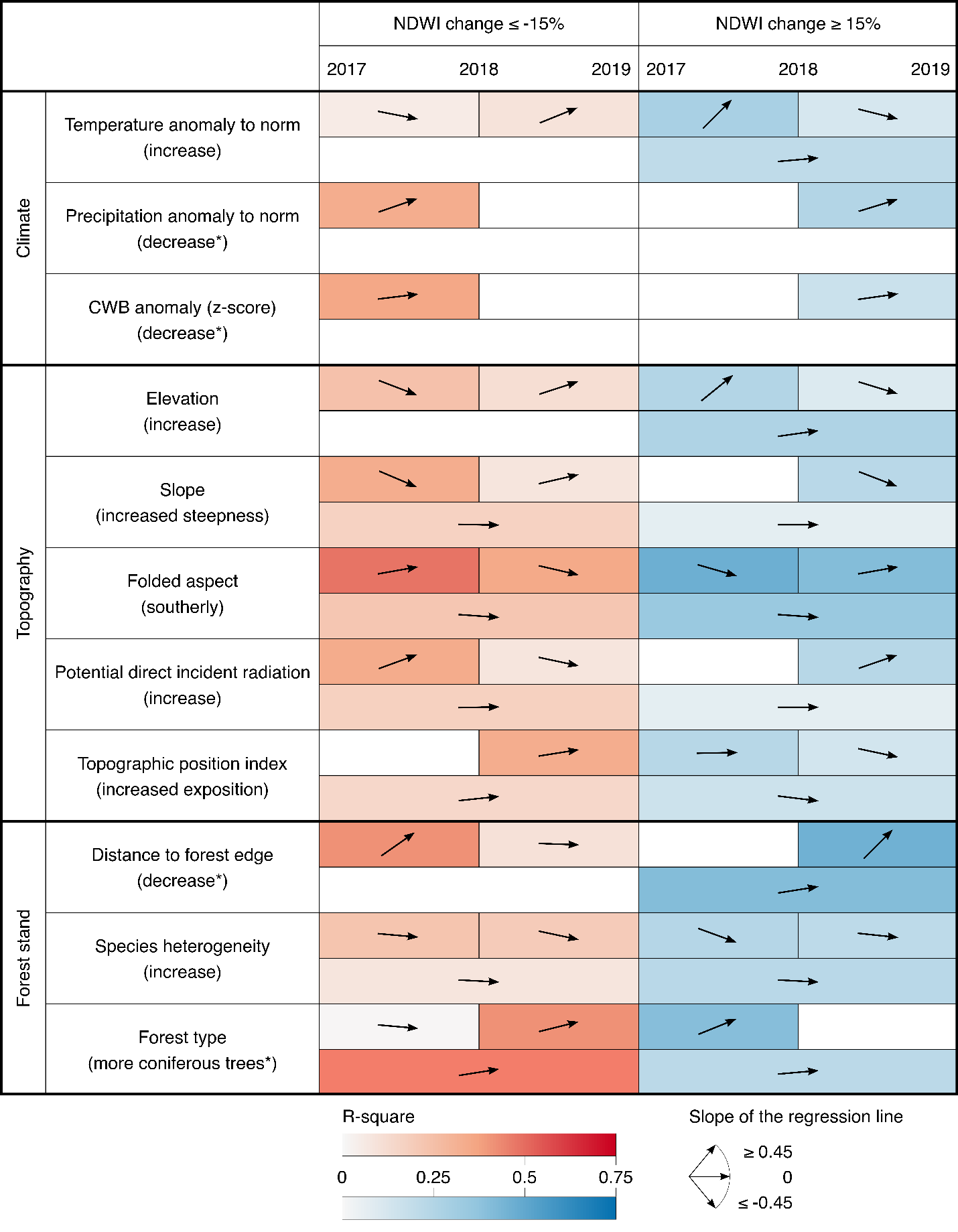


Figure S7: Linear models for environmental variables and positive or negative NDWI changes of ≥ 15% for 2017–2018 (resistance to the 2018 drought event), 2018–2019 (recovery), and 2017–2019 (resilience). The R^2^ values of each relation are colored either in red (NDWI change ≤ -15%) or blue (NDWI change ≥ 15%). The arrows indicate the slopes of the linear regressions (using the x-axis with intervals from 1–10: changes in percentage affected pixels across the 10 intervals of each environmental variable). For the variables marked with a * the regression slopes are inverse to the range presented in Table 1 (max–min rather than min–max)­ to focus on drought characteristics (climate) or site characteristics where higher proportions of damaged forest were found (forest stand). The intensity of color represents the effect size as measured by the R^2^ value of the regression. Fields of non-significant relations (*p* > 0.05) are empty.


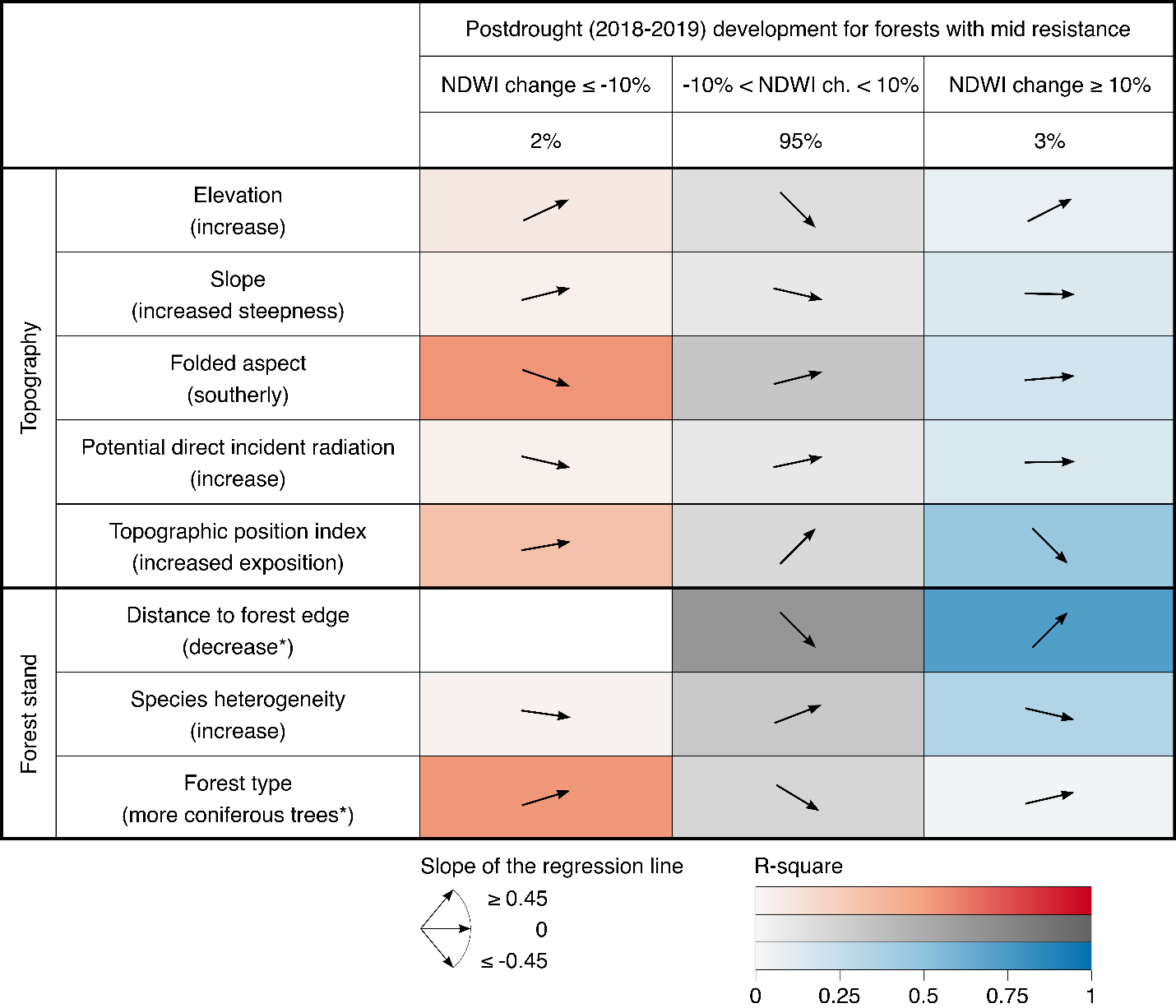


Figure S8: Forest recovery quantified as ≥ 10% change of NDWI from 2018 to 2019, for all forest areas with NDWI change < 10% from 2017 to 2018, i.e. mid resistance. The R^2^ values of each relation are colored either in red for areas with further decreasing NDWI (negative NDWI change ≥ 10% from 2018–2019, i.e. negative resistance; 2% of cases), grey for areas with little further change in NDWI (change from 2018–2019 less than 10%; 95% of cases), or blue for areas recovering from low resistance (positive NDWI change ≥ 10% from 2018–2019, i.e. positive recovery; 3% of cases). The arrows indicate the slopes of the linear regressions between the proportion of forest areas with NDWI change and the affecting environmental variable (using the x-axis with intervals from 1-10: changes in percentage affected pixels across the 10 intervals or each environmental variable; for the first column slopes are multiplied by 10 because here changes are small in relation to the percentage values). For the variables marked with a * the regression slopes are inverse to the range presented in Table 1 (max–min rather than min–max) to focus on the site characteristics where higher proportions of damaged forest were found. Fields of non-significant relations (*p* > 0.05) are empty.


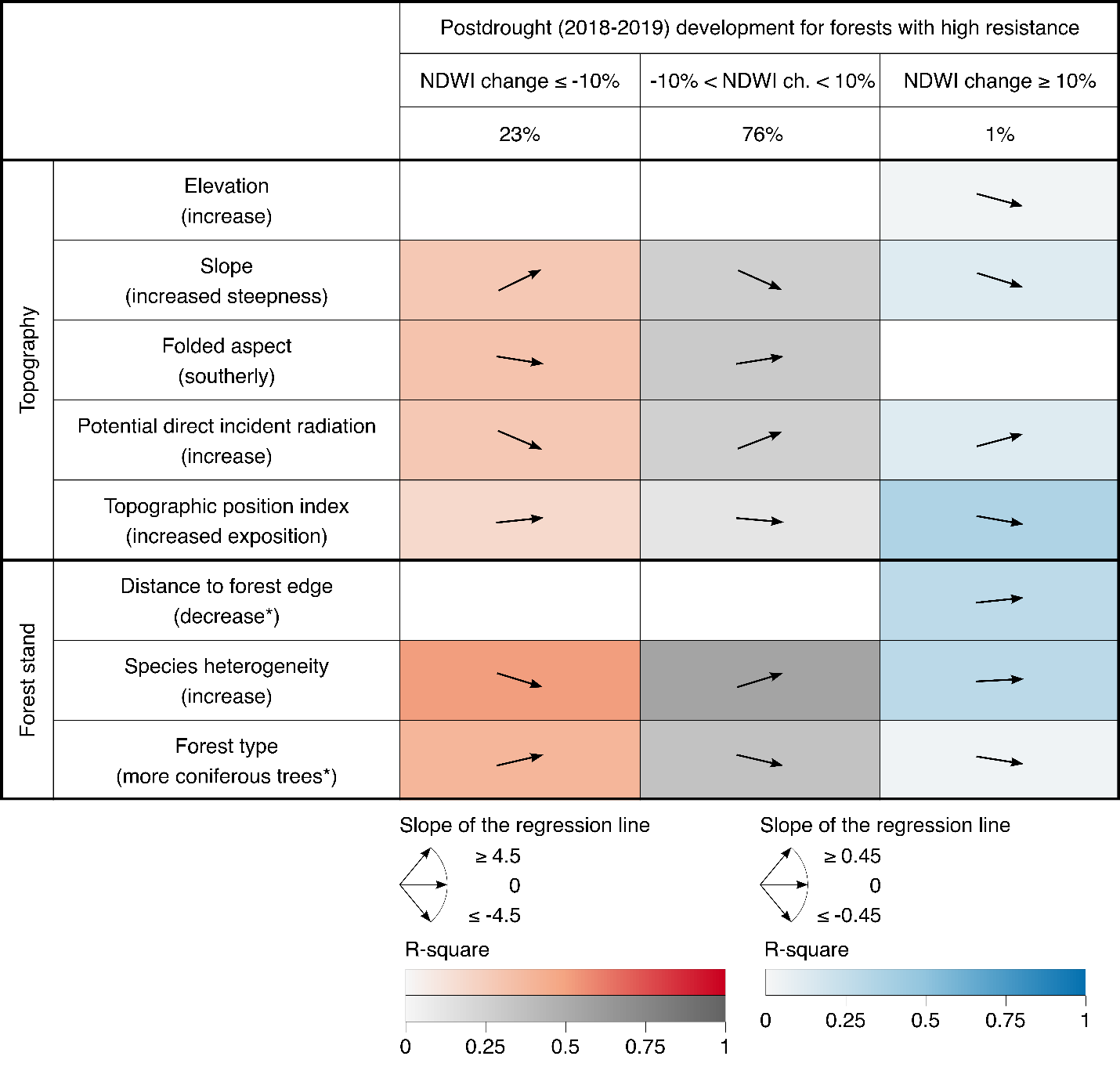


Figure S9: Forest recovery quantified as ≥ 10% change of NDWI from 2018 to 2019, for all forest areas with a positive NDWI change ≥ 10% from 2017 to 2018, i.e. high resistance. The R^2^ values of each relation are colored either in red for areas with further decreasing NDWI (negative NDWI change ≥ 10% from 2018–2019, i.e. negative resistance; 23% of cases), grey for areas with little further change in NDWI (change from 2018–2019 less than 10%; 76% of cases), or blue for areas recovering from low resistance (positive NDWI change ≥ 10% from 2018–2019, i.e. positive recovery; 1% of cases). The arrows indicate the slopes of the linear regressions between the proportion of forest areas with NDWI change and the affecting environmental variable (using the x-axis with intervals from 1-10: changes in percentage affected pixels across the 10 intervals or each environmental variable; for the first column slopes are multiplied by 10 because here changes are small in relation to the percentage values). For the variables marked with a * the regression slopes are inverse to the range presented in Table 1 (max–min rather than min–max) to focus on the site characteristics where higher proportions of damaged forest were found. Fields of non-significant relations (*p* > 0.05) are empty.

| **NDWI change ≤ –10%** | | | **Resistance** | | **Recovery** | | **Resilience** | |
| --- | --- | --- | --- | --- | --- | --- | --- | --- |
|  | numDF | denDF | F-value | p-value | F-value | p-value | F-value | p-value |
| (Intercept) | 1 | 2048 | 418.3 | <.0001 | 279.7 | <.0001 | 250.6 | <.0001 |
| ELE | 2 | 2048 | 192.2 | <.0001 | 192.9 | <.0001 | 82.5 | <.0001 |
| ASP | 2 | 2048 | 306.9 | <.0001 | 790.1 | <.0001 | 99.3 | <.0001 |
| TOP | 2 | 2048 | 4 | 0.0183 | 8.7 | 0.0002 | 15.2 | <.0001 |
| DIS | 2 | 2048 | 2045.9 | <.0001 | 26.7 | <.0001 | 71.8 | <.0001 |
| COM | 2 | 2048 | 436.5 | <.0001 | 438.2 | <.0001 | 730 | <.0001 |
| ELE:ASP | 4 | 2048 | 23.9 | <.0001 | 54.8 | <.0001 | 19.1 | <.0001 |
| ELE:TOP | 4 | 2048 | 9.3 | <.0001 | 1.5 | 0.2042 | 2.2 | 0.0696 |
| ELE:DIS | 4 | 2048 | 224.9 | <.0001 | 2.6 | 0.0353 | 25.6 | <.0001 |
| ELE:COM | 4 | 2048 | 5.2 | 0.0004 | 20.7 | <.0001 | 16.8 | <.0001 |
| ASP:TOP | 4 | 2048 | 3.3 | 0.0109 | 7.7 | <.0001 | 2.8 | 0.0233 |
| ASP:DIS | 4 | 2048 | 12.3 | <.0001 | 0.7 | 0.5606 | 1.6 | 0.1758 |
| ASP:COM | 4 | 2048 | 3.8 | 0.0041 | 30.4 | <.0001 | 32.5 | <.0001 |
| TOP:DIS | 4 | 2048 | 32.2 | <.0001 | 0.9 | 0.4633 | 6.9 | <.0001 |
| TOP:COM | 4 | 2048 | 1.5 | 0.1857 | 4.1 | 0.0027 | 1.2 | 0.3192 |
| DIS:COM | 4 | 2048 | 26.3 | <.0001 | 1.3 | 0.2585 | 5.2 | 0.0004 |
| **NDWI change ≥ 10%** | | | **Resistance** | | **Recovery** | | **Resilience** | |
|  | numDF | denDF | F-value | p-value | F-value | p-value | F-value | p-value |
| (Intercept) | 1 | 2048 | 94.2 | <.0001 | 319.1 | <.0001 | 169.2 | <.0001 |
| ELE | 2 | 2048 | 496.4 | <.0001 | 343.2 | <.0001 | 435.6 | <.0001 |
| ASP | 2 | 2048 | 865.7 | <.0001 | 388 | <.0001 | 127 | <.0001 |
| TOP | 2 | 2048 | 4.3 | 0.0143 | 61.5 | <.0001 | 107.9 | <.0001 |
| DIS | 2 | 2048 | 28.5 | <.0001 | 2795.9 | <.0001 | 222.4 | <.0001 |
| COM | 2 | 2048 | 424.7 | <.0001 | 572 | <.0001 | 471.3 | <.0001 |
| ELE:ASP | 4 | 2048 | 49.9 | <.0001 | 64.8 | <.0001 | 24.8 | <.0001 |
| ELE:TOP | 4 | 2048 | 2 | 0.0995 | 7.5 | <.0001 | 1.6 | 0.1637 |
| ELE:DIS | 4 | 2048 | 2.8 | 0.0258 | 249.3 | <.0001 | 7.8 | <.0001 |
| ELE:COM | 4 | 2048 | 83 | <.0001 | 28.8 | <.0001 | 86.2 | <.0001 |
| ASP:TOP | 4 | 2048 | 5.1 | 0.0004 | 4.7 | 0.0009 | 2 | 0.0869 |
| ASP:DIS | 4 | 2048 | 1.1 | 0.3764 | 14.5 | <.0001 | 2 | 0.0982 |
| ASP:COM | 4 | 2048 | 24.3 | <.0001 | 19.2 | <.0001 | 40.5 | <.0001 |
| TOP:DIS | 4 | 2048 | 0.3 | 0.9080 | 30.5 | <.0001 | 2.5 | 0.0433 |
| TOP:COM | 4 | 2048 | 5.5 | 0.0002 | 1.5 | 0.1914 | 2.1 | 0.0821 |
| DIS:COM | 4 | 2048 | 1.1 | 0.3574 | 20.3 | <.0001 | 0.7 | 0.6074 |

Table S1: Table with the ANOVA output of the linear mixed-effects model of the factors ELE (elevation: 0–800 m; 801–1600 m; >1600 m a.s.l.), ASP (folded aspect: 0–60°; 61–120°; 121–180°), TOP (the topographic position index: < –1 standard deviation (valleys); ≥ –1 and ≤ 1 standard deviation; > 1 standard deviation (ridges) (Weiss, 2001)) , DIS (the distance to the forest edge: 0–20 m; 21–50m; >50 m), and COM (the forest type: 0–20 % (coniferous); 21–79% (mixed); 80–100% (broadleaf)) in which ten biogeographic regions were fitted as random-effects term. numDF: degree of freedom of numerator, i.e. term fitted as 3-level factor or interaction of two factors, denDF: denominator degree of freedom (this was constant because the random-effects term was not confounded with the fixed terms).

| **Mean NDWI change** | | | **Resistance** | | **Recovery** | | **Resilience** | |
| --- | --- | --- | --- | --- | --- | --- | --- | --- |
|  | numDF | denDF | F-value | p-value | F-value | p-value | F-value | p-value |
| (Intercept) | 1 | 2048 | 0.1 | 0.7497 | 13.2 | 0.0003 | 0.8 | 0.3836 |
| ELE | 2 | 2048 | 443.9 | <.0001 | 603.3 | <.0001 | 194.0 | <.0001 |
| ASP | 2 | 2048 | 520.0 | <.0001 | 677.8 | <.0001 | 7.2 | 0.0007 |
| TOP | 2 | 2048 | 8.6 | 0.0002 | 46.6 | <.0001 | 98.7 | <.0001 |
| DIS | 2 | 2048 | 1143.0 | <.0001 | 1545.4 | <.0001 | 0.8 | 0.4300 |
| COM | 2 | 2048 | 101.3 | <.0001 | 123.7 | <.0001 | 200.5 | <.0001 |
| ELE:ASP | 4 | 2048 | 3.5 | 0.0078 | 17.2 | <.0001 | 4.1 | 0.0025 |
| ELE:TOP | 4 | 2048 | 6.3 | <.0001 | 3.8 | 0.0043 | 5.9 | 0.0001 |
| ELE:DIS | 4 | 2048 | 129.2 | <.0001 | 127.0 | <.0001 | 12.9 | <.0001 |
| ELE:COM | 4 | 2048 | 24.2 | <.0001 | 27.8 | <.0001 | 32.2 | <.0001 |
| ASP:TOP | 4 | 2048 | 5.8 | 0.0001 | 6.6 | <.0001 | 0.7 | 0.5767 |
| ASP:DIS | 4 | 2048 | 11.3 | <.0001 | 13.2 | <.0001 | 0.2 | 0.9264 |
| ASP:COM | 4 | 2048 | 7.7 | <.0001 | 15.4 | <.0001 | 10.8 | <.0001 |
| TOP:DIS | 4 | 2048 | 18.7 | <.0001 | 13.1 | <.0001 | 3.3 | 0.0103 |
| TOP:COM | 4 | 2048 | 4.2 | 0.0022 | 6.3 | <.0001 | 1.8 | 0.1234 |
| DIS:COM | 4 | 2048 | 21.7 | <.0001 | 20.8 | <.0001 | 5.7 | 0.0001 |

Table S2: Table with the ANOVA output of the linear mixed-effects model of the factors ELE (elevation: 0–800 m; 801–1600 m; >1600 m a.s.l.), ASP (folded aspect: 0–60°; 61–120°; 121–180°), TOP (the topographic position index: < –1 standard deviation (valleys); ≥ –1 and ≤ 1 standard deviation; > 1 standard deviation (ridges) (Weiss, 2001)) , DIS (the distance to the forest edge: 0–20 m; 21–50m; >50 m), and COM (the forest type: 0–20 % (coniferous); 21–79% (mixed); 80–100% (broadleaf)) in which ten biogeographic regions were fitted as random-effects term. numDF: degree of freedom of numerator, i.e. term fitted as 3-level factor or interaction of two factors, denDF: denominator degree of freedom (this was constant because the random-effects term was not confounded with the fixed terms).
